# Revision of the genus *Paridea* Baly, 1886 from Taiwan (Coleoptera, Chrysomelidae, Galerucinae)

**DOI:** 10.3897/zookeys.405.7458

**Published:** 2014-04-28

**Authors:** Chi-Feng Lee, Jan Bezděk

**Affiliations:** 1 Applied Zoology Division, Taiwan Agricultural Research Institute, 189 Chung-Cheng Road, Wufeng, Taichung 413, Taiwan; 2 Department of Zoology, Mendel University, Zemědělská 1, 613 00 Brno, Czech Republic

**Keywords:** *Paridea*, *Semacia*, Taiwan, taxonomic revision

## Abstract

The Taiwanese species of the genus *Paridea* Baly, 1886, are revised. Two new species, *Paridea (Semacia) houjayi* **sp. n.** and *P. (S.) kaoi* **sp. n.** are described. Both were confused previously with *P. (S.) sexmaculata* (Laboissière, 1930) and *P. (S.) angulicollis* (Motschulsky, 1854) respectively. *Paridea (P.) sauteri* (Chûjô, 1935) and *P. (P.) taiwana* (Chûjô, 1935) are removed from synonymy with *P. (P.) sinensis* Laboissière, 1930. The synonymy of *Paraulaca flavipennis* Chûjô, 1935 with *Paridea (Paridea) testacea* Gressitt & Kimoto, 1963 is supported. *Paridea (Semacia) nigrimarginata* Yang, 1991 is regarded as a junior synonym of *P. (S.) angulicollis* and excluded from the Taiwan fauna. Lectotypes are designated for *Paraulaca costata* Chûjô, 1935, *P. flavipennis* Chûjô, 1935, *P. taiwana* Chûjô, 1935, *Semacia nipponensis* Laboissière, 1930, and *Paridea sinensis* Laboissière, 1930.

## Introduction

The genus *Paridea* Baly, 1886, comprising 83 species (Bezděk, unpublished data), is one of the most species-rich genera of Oriental Galerucinae. The genus occurs in Nepal, Bhutan, India, Pakistan, China, Korea, Japan and Southeast Asia. *Paridea apicalis* (Jacoby, 1886), from New Guinea, evidently is not congeneric and is not counted. As in most large Oriental genera of Galerucinae, a comprehensive revision based on the study of primary types has not been performed. Recently, only two larger papers were devoted to *Paridea*: the Chinese species were revised by Yang (1991) and Medvedev and Samoderzhenkov (1997) provided a key to species from the Himalayas and adjacent regions.

*Paridea* Baly, 1886 is similar to other genera of the subtribe Aulacophorina, particularly to *Agetocera* Hope, 1831, *Aulacophora* Chevrolat, 1836, *Pseudocophora* Jacoby, 1884 and *Paragetocera* Laboissière, 1929. All these genera share unmargined anterior pronotal margins, the presence of a transverse depression on the pronotum, and all feed on leaves of Cucurbitaceae (cf. Kimoto 1989b, Jolivet and Hawkeswood 1995). *Paridea* is separated easily from other genera by the appendiculate tarsal claws (bifid tarsal claws in others).

Laboissière (1930) described the first *Paridea* species from Taiwan as *Semacia sexmaculata* Laboissière. Chûjô (1935) added five species to the Taiwan fauna as *Paraulaca costata* Chûjô, *Paraulaca cyanipennis* Chûjô, *Paraulaca flavipennis* Chûjô, *Paraulaca sauteri* Chûjô, and *Paraulaca taiwana* Chûjô. Chûjô (1938) later listed a newly recorded species, *Paraulaca angulicollis* (Motschulsky, 1854). Kimoto (1966) synonymized *Paraulaca taiwana* Chûjô, 1935 with *Paraulaca sauteri* Chûjô, 1935; *Paraulaca flavipennis* Chûjô, 1935 with *Paridea testacea* Gressitt & Kimoto, 1963. *Paridea testacea* remains valid because *Paraulaca flavipennis* Chûjô, 1935 is a secondary homonym of *Paraulaca flavipennis* (Laboissière, 1930). Kimoto (1974) regarded *Paraulaca sauteri* Chûjô, 1935 as a junior synonym of *Paridea sinensis* Laboissière, 1930. Yang (1991) studied the Chinese species of *Paridea* and described a new species, *Paridea nigrimarginata* from Mt. Takao. Beenen (2010) added this species to Taiwan fauna since he assumed the type locality is in Taiwan. Thus, in total seven species have been described or recorded from Taiwan.

Taxonomic confusion and misidentification often occurs in this genus because some members have variable color patterns and some others have sexually dimorphic characters. Thus, robust sample sizes are required for revising this genus. The Taiwan Chrysomelid Research Team (TCRT) was founded in 2005 and is composed of 10 members. All of them are amateurs interested in making an inventory of all Chrysomelid species in Taiwan. Specimens of the genus have been extensively collected and studied, and host plants recorded. Diagnostic characters were assessed and the status of all species was evaluated based on a large series of specimens.

## Materials and methods

More than 1000 specimens have been examined. Most of them (> 90%) were collected either by TCRT or belonged to the historical collection at the Taiwan Agricultural Research Institute (TARI).

To prepare drawings of the adult reproductive systems, the abdomens of adults were separated and boiled in a 10% KOH solution, cleared in distilled water, and then mounted on microscope slides in glycerin for observation. Specimens were examined and drawings were made using a Leica M165 stereomicroscope. Microscope slides were examined and illustrated using a Nikon ECLIPSE 50i microscope. Body parts were then stored in glycerin tubes with the dry mounted specimens.

Host plants are recorded by observing adult feeding behavior in the field. Plants were identified by Chih-Kai Yang.

Specimens examined are deposited at the following institutes and museums.

BMNH The Natural History Museum, London, UK [Michael Geiser];

BPBM Bernice P. Bishop Museum, Hawaii, USA [Shepherd Myers];

CAS California Academy of Sciences, California, USA [David H. Kavanaugh];

FKCC František Kantner collection, České Budějovice, Czech Republic;

HNHM Hungarian Natural History Museum, Budapest, Hungary [Otto Merkl];

ISNB Institut Royal des Sciences Naturelles de Belgique, Brussels, Belgium [Pol Limbourg];

IZAS Institute of Zoology, Academia Sinica, Beijing, China [YongYing Ruan];

JBCB Jan Bezděk collection, Brno, Czech Republic;

KMNH Kitakyushu Museum of Natural History, Kitakyushu, Japan [Kyoichiro Ueda];

MNHN Museum National d’Histoire naturelle, Paris, France [Antoine Mantilleri];

NMNS National Museum of Natural Science, Taichung, Taiwan [Ming-Luen Jeng];

SDEI Senckenberg Deutsches Enomologisches Institut, Müncheberg, Germany [Stephan Blank];

TARI Taiwan Agricultural Research Institute, Taichung, Taiwan;

ZMUH Zoologisches Institut und Zoologisches Museum, Universität von Hamburg, Hamburg, Germany [Hossein Rajaei and Kai Schuette];

Exact label data are cited for all type specimens of the described species; a double slash (//) divides the data on different labels and a single slash (/) divides the data in different rows. Other comments and remarks are in square brackets: [p] – preceding data are printed, [h] – preceding data are handwritten, [w] – white label, [y] – yellow label, [o] – orange label, [g] – green label, and [r] – red label.

## Taxonomy

### Classification of subgenera of *Paridea*

Five generic or subgeneric names (*Paraulaca* Baly, 1888, *Aeropa* Weise, 1889, *Semacia* Fairmaire, 1889, *Semacianella* Laboissière, 1930, and *Carapaula* Chûjô, 1962) were erected based on various sexually dimorphic characters of *Paridea*. Kimoto (1989b) and Medvedev and Samoderzhenkov (1997) used *Paridea* (s. str.), *Paraulaca* and *Semacia* as valid subgenera, while Yang (1991) considered only *Paridea* and *Semacia* as monophyletic after reviewing Chinese species. This arrangement was followed also by Beenen (2010) (with *Paraulaca*, *Carapaula* and *Semacianella* as synonyms of *Paridea* (s. str.) and *Aeropa* as synonym of *Semacia*) and is used also in our study but some characters are modified in the key

#### Key to species of the genus *Paridea* from Taiwan

**Table:** 

1	Pygidium of both sexes projecting beyond elytral apices; apex of eighth abdominal tergite in male modified into two processes, apical margin without setae	2 (subgenus *Semacia*)
–	Pygidium of both sexes covered by elytra; apex of eighth abdominal tergite not modified, apical margin with setae	4 (subgenus *Paridea*)
2	Female pygidium deeply emarginated (Fig. 4); elytron of male with one premedian cavity near lateral margin (Fig. 3); without black spot on postscutellar common area in female (Fig. 4)	*Paridea (Semacia) houjayi* sp. n.
–	Female pygidium entire; elytron of male without a cavity near lateral margin but with postscutellar area depressed (Figs 6, 28, 31); flat and with black spot in female (Figs 9, 32, 33)	3
3	Elytron with longitudinal black stripe along lateral margin from base to middle (Fig. 8)	*Paridea (Semacia) kaoi* sp. n.
–	Elytron without black stripe along lateral margin, but with one subbasal black spot near lateral margin (Fig. 30), sometimes extending inwards (Figs 31, 33)	*Paridea (Semacia) sexmaculata* (Laboissière)
4	Elytron at least partly metallic blue or black	5
–	Elytron yellowish brown, sometimes with two pairs of black spots, in some individuals black spots extending to entire elytron except apex	6
5	Vertex black; pronotum with one black spot at middle near basal margin; first tarsomeres of front and middle legs in male strongly swollen (Fig. 34); entire elytra metallic blue (Figs 34, 36)	*Paridea (Paridea) costata* (Chûjô)
–	Head and prontum yellowish brown; first tarsomeres in male not modified (Fig. 52); lateral margin of elytron from base to apical 1/3 yellowish brown (Fig. 54)	*Paridea (Paridea) cyanipennis* (Chûjô)
6	Femora and tibia yellowish brown, but outer margins black (Figs 74–79)	*Paridea (Paridea) taiwana* (Chûjô)
–	Femora yellowish; tibiae blackish brown or black	7
7	Elytra yellowish brown (Fig. 80)	*Paridea (Paridea) testacea* Gressitt & Kimoto
–	Elytra black, but apices yellowish brown (Fig. 55)	*Paridea (Paridea) sauteri* (Chûjô)

### Paridea (Semacia) houjayi sp. n.

http://zoobank.org/6D9F8E80-A589-489A-A9A0-E0129A38E5E8

http://species-id.net/wiki/Paridea_houjayi

Paridea (Paraulaca) sexmaculata : Kimoto & Takizawa, 1997: 180 (misidentification)

#### Type locality.

Taiwan, Ilan, Ssuchi, 24°29’N, 121°25E, 700 m, broad-leaf forest.

#### Type material

**(n = 16).** Holotype male (TARI), mounted on card, labeled: “Taiwan: Ilan (12458) [p] / Ssuchi (四季) [p] / 01.VIII.2009, leg. M.-H. Tsou [p, w] // **Holotypus** [p] / *Paridea (Semacia)* ♂ [p] / *houjayi* n. sp. [p] / des. C.-F. Lee, 2014 [p, r]”. Paratypes: 4♀♀, same data as holotype, but with “12459–12462” (TARI); 1♀: “Taiwan: Hsinchu (8723) [p] / Lupi (魯壁) [p] / 12.III.2009, leg. H. Lee [p, w]” (TARI); 1♂: “Taiwan: Ilan (4098) [p] / Fushan Chihwuyuan [= Botanical Park] [p] / 01.IV.2008, leg. M.-H. Tsou [p, w]” (TARI); 1♂: “Hatonosawa [p] [= Chiuchihtse] / Mt. Taiheizan [p] [= Taipingshan] / 23.vii.1940 [p] / FORMOSA [p] / Col. M. CHUJO [p, w]” (TARI); 1♀: “Taiwan: Ilan (4712) [p] / Mingchi (明池) [p] / 27.IV.2008, leg. M.-H. Tsou [p, w]” (TARI); 1♂: “N. TAIWAN: Taipingshan [p] / 1950m. Ilan Hsien [p] / 26–28.VII.1983 [p] / L. Y. Chou [p, o]” (TARI); 1♂: “Taiwan: Taipei (4772) [p] / Fushan [p] / 30.IV.2008, leg. H.-J. Chen [p, w]” (TARI); 1♀: “Taiwan: Taipei (18945) [p] / Fushan (福山) [p] / 01.IV.2011, leg. S.-F. Yu [p, w]” (TARI); 1♂: “Taiwan: Taipei (989) [p] / Wulai [p] / 15.III.2007, leg. H.-J. Chen [p,w]” (TARI); 1♂: “Taiwan: Taipei [p] / Wulai [p] / 05.III.2009, leg. Y.-L. Lin [p,w]” (TARI); 1♀: “Taiwan: Taoyuan (3980) [p] / Hsuanyuan (萱源) [p] / 16.III.2008, leg. M.-H. Tsou [p,w]” (TARI); 1♂: “Taiwan: Taoyuan (4691) [p] / Tungyanshan (東眼山) [p] / 25.IV.2008, leg. H. Lee [p, w]” (TARI); 1♂: “TAIWAN, Ilan county, [p] / Mingchyh Forest [p] / Recreation Area, 1200 m, [p, w] // swept from vegetation, [p] / 5.IV.2002, [p] / leg. Gy. Fábián & O. Merkl [p, w]” (HNHM). Each paratype has a type label: “**Paratypus** [p] / *Paridea (Semacia)* ♂ [or ♀] [p] / *houjayi* n. sp. [p] / des. C.-F. Lee, 2014 [p, pink label]”.

#### Diagnosis.

This new species is recognized easily by the elytra of males possessing a lateral cavity near each lateral margin and the pygidium of each female with deeply emarginated apex.

#### Males.

**Figures 1–9.**
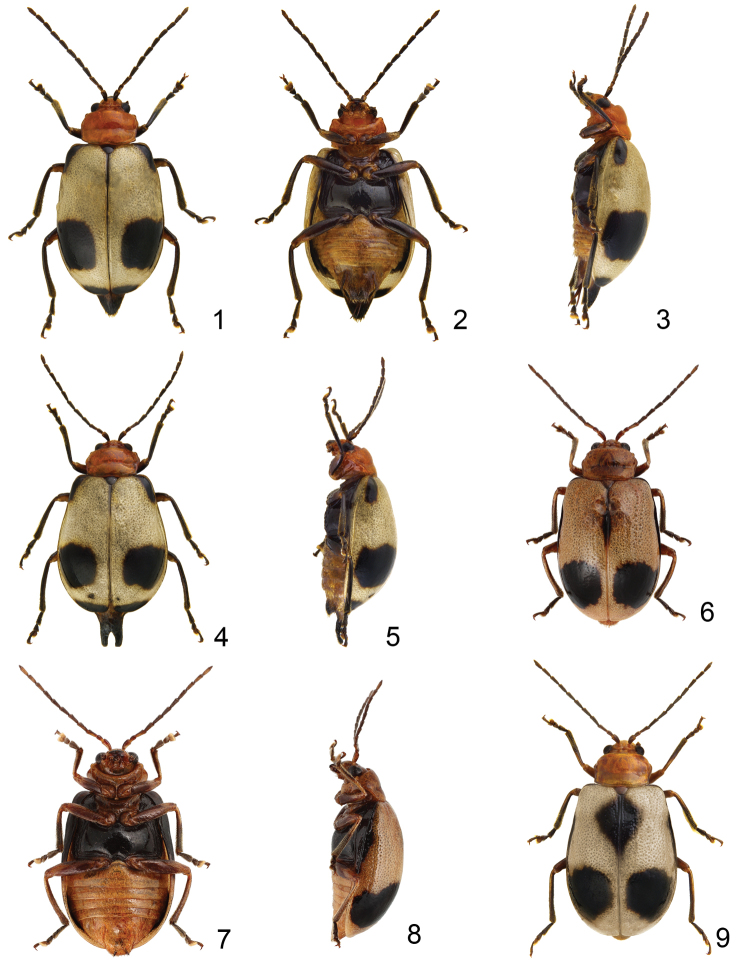
*Paridea* species. **1** *Paridea (Semacia) houjayi* sp. n., male, dorsal view **2** ditto, ventral view **3** ditto, lateral view **4** *Paridea (Semacia) houjayi* sp. n., female, dorsal view 5 ditto, lateral view **6** *Paridea (Semacia) kaoi* sp. n., male, dorsal view **7** ditto, ventral view **8** ditto, lateral view **9** *Paridea (Semacia) kaoi* sp. n., female, dorsal view.

**Figures 10–16.**
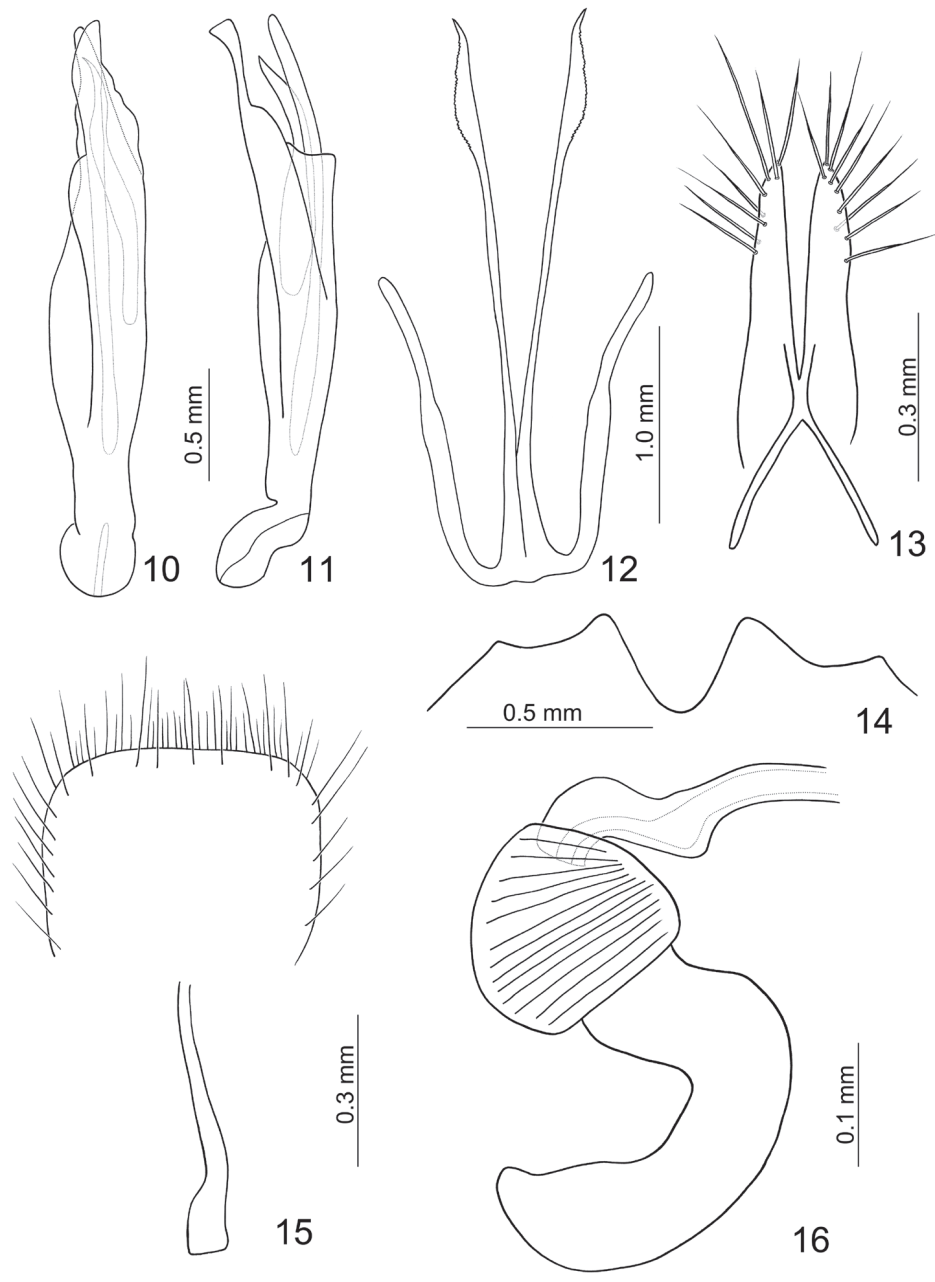
*Paridea (Semacia) houjayi* sp. n. **10** Penis, dorsal view **11** Penis, lateral view **12** Eighth abdominal tergite **13** Gonocoxae **14** Fifth abdominal ventrite **15** Eighth abdominal sternite **16** Spermatheca.

Length 6.2–6.3 mm, width 3.6–3.7 mm. General color (Figs 1–3) yellowish brown; antenna dark brown; scutellum black; elytra pale yellow, with one small, longitudinal black spot at humerus, one large, rounded black spot at postermedian area, apex black; mesepimeron and metathoracic ventrites black; outer margins of femora and tibiae black; tarsi dark brown. Elytron with one premedian cavity near lateral margin; three tufts of long hairs at anterior area and one tubercle at posterior of depression; postscullar common area flat and impunctate. Eighth abdominal tergite (Fig. 12) strongly sclerotized, base extremely slender, with one pair of extremely long processes, each process flattened and widened near apex and outer margin of widened area serrate. Pygidium projecting beyond elytral apices, apex shallowly emarginate. Penis (Figs 10–11) strongly asymmetric, dorsum with longitudinal groove at right side; almost straight from lateral view; apex forming angular process and directed ventrally; endophallic sclerites with two sclerites, one extremely elongate, about 0.75 times as long as penis, other curved and apically pointed.

#### Females.

Length 6.2–6.9 mm, width 3.5–3.7 mm. Color (Figs 4–5) similar to male but elytra without excavation. Apical margin of last abdominal ventrite (Fig. 14) with deep notch at middle, between one pair of shallow processes. Pygidium projecting beyong elytral apices, deeply emarginate and forming bilobed process. Gonocoxae (Fig. 13) slender, apex of each gonocoxa with eight to nine setae from apical 1/4 to apex; connection of gonocoxae extremely slender, base slender. Sternite VIII (Fig. 15) weakly sclerotized; apex wide, apical margin truncate, surface with extremely dense long setae along apical margin, spiculum short. Spermathecal receptaculum (Fig. 16) strongly swollen; pump much longer than receptaculum, strongly curved; spermathecal duct short, stout, shallowly projecting into receptaculum.

#### Etymology.

The new species name honors Mr. Hou-Jay Chen, who discovered this interesting new species.

#### Distribution.

**Figures 17–20.**
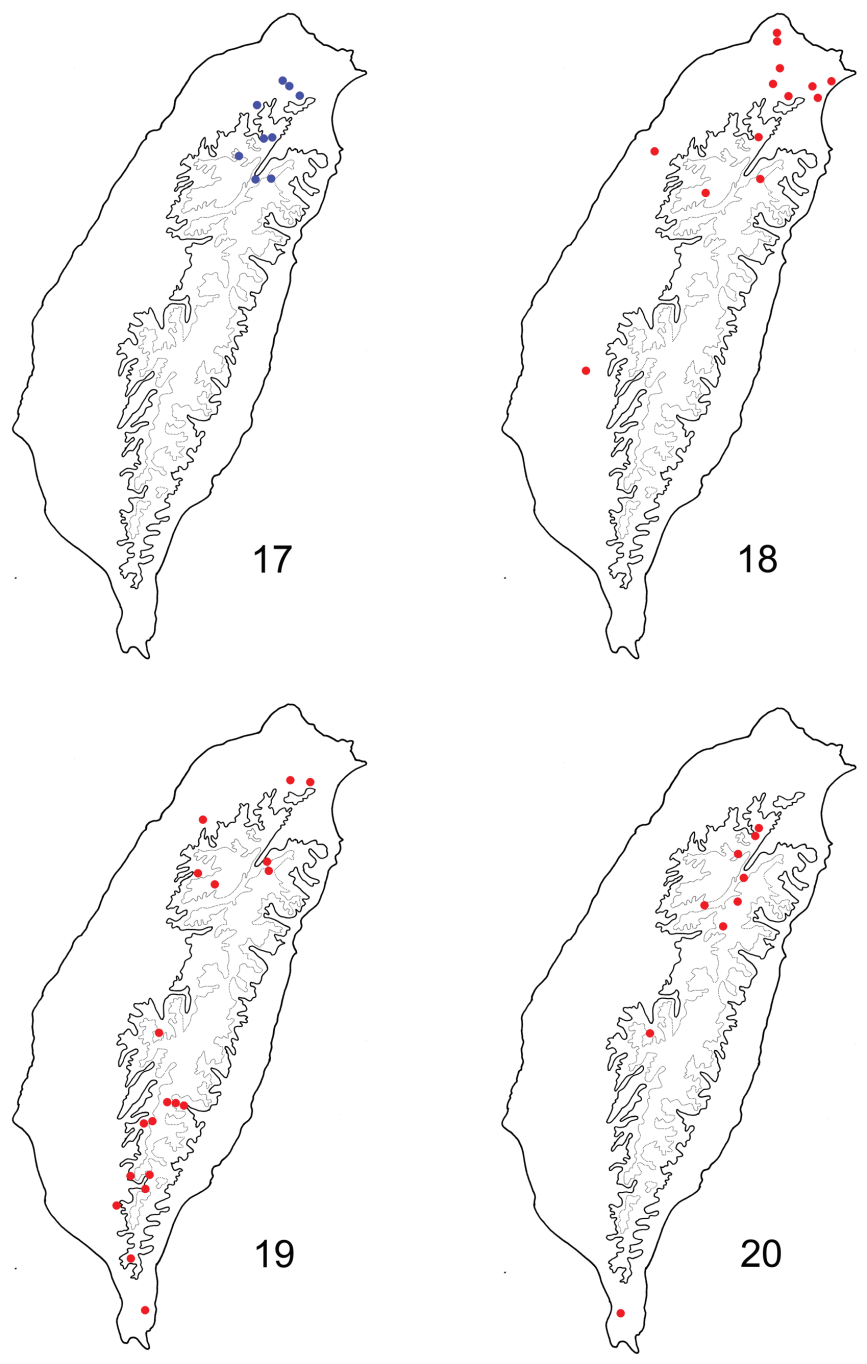
Distribution map of *Paridea* species, solid line: 1000 m, broken line: 2000 m. **17** *Paridea (Semacia) houjayi* sp. n. **18** *Paridea (Semacia) kaoi* sp. n. **19** *Paridea (Semacia) sexmaculata* **20** *Paridea (Paridea) costata*.

Taiwan. This new species occurs in northern Taiwan (Fig. 17).

#### Host plants.

Cucurbitaceae: *Gynostemma pentaphyllum* (Thunb.) Makino; *Thladiantha nudiflora* Hemsl. ex Forbes & Hemsl.; Compositae: *Aster lasiocladus* Hayata.

#### Notes.

Males of this new species were misidentified as *Paridea (Semacia) sexmaculata* by Kimoto and Takizawa (1997).

### Paridea (Semacia) kaoi sp. n.

http://zoobank.org/9FB73A3A-10B1-4603-98D0-D2D8523F37AC

http://species-id.net/wiki/Paridea_kaoi

Paraulaca angulicollis : Chûjô 1938: 138 (misidentification)
Paraulaca (Paraulaca) angulicollis : Chûjô 1962: 195 (redescription); Chûjô 1963: 395.

#### Type locality.

Taiwan, Taipei, Wulai, 24°51’N, 121°33E, 150 m, broad-leaf forest.

#### Type material

**(n = 27).** Holotype male (TARI), mounted on card, labeled: Taiwan: Taipei (130) [p] / Wulai [p] / 05.IX.2006, leg. H.-T. Cheng [p, w] // **Holotypus** [p] / *Paridea (Semacia)* ♂ [p] / *kaoi* n. sp. [p] / des. C.-F. Lee, 2014 [p, r]”. Paratypes: 1♀: “Taihorinsho [h] / Formosa [p] / Sauter [p] IX [h] __ 07 09 [p, w] // *Paraulaca* [h] / angulicollis [h] / Motschulsky [h] / DET. M. CHUJO [p, g] // DEI Müncheberg [p] / Col – 04198 [p, g]” (SDEI); 1♀: “LB [h] 973 [h, w, circular label] / Taiwan: Taipei (973) [p] / Wulai [p] / 15.III.2007, leg. H.-J. Chen [p,w]” (TARI); 3♂♂: “Taiwan: Taipei, [p] / Wulai, 30.III.2007 [p] / leg. C.-F. Lee [p, w]” (TARI); 1♀: “Taiwan: Taipei (10866) [p] / Wulai [p] / 22.V.2009, leg. H.-J. Chen [p,w]” (TARI); 1♀: “Taiwan: Taipei (11678) [p] / Wulai [p] / 26.VI.2009, leg. H.-J. Chen [p, w]” (TARI); 1♂: “TAIWAN: Wulai [p] / nr. Taipei, 300- [p] / 500m, 23.IX.1957 [p, w] // K.S. Lin [p] / Collector [p] / BISHOP“ (BPBM); 1♂: “TAIWAN (NE): [p] / Taiheizan [= Taipingshan], 1500m [p] / 6 [h]. VI/1934 [p] / L. Gressitt [p, w]“ (BPBM); 1♂: “Taiwan: Ilan (5258) [p] / Fushan Chiwuyuan [p] [= Botanical Park] / 08.V.2008, leg. M.-H. Tsou [p,w]” (TARI); 1♂: “Taiwan: Ilan [p] / Fushan (福山)植物園 [p] / 13.IV.2011, leg. C.-F. Lee [p,w]” (TARI); 1♂: “Taiwan: Ilan (4711) [p] / Mingchi (明池) [p] / 27.IV.2008, leg. M.-H. Tsou [p, w]” (TARI); 1♀: “Taiwan: Ilan [p] / Yingtzuling (鶯仔嶺) [p] / 15.IV.2012, leg. Y.-L. Lin [p,w]” (TARI); 1♂: “Taiwan: Miaoli (4776) [p] / Hohsinglinchang (和興林場) [p] / 01.V.2008, leg. Y.-C. Lin [p,w]” (TARI); 1♂: “Taiwan: Taichung (3193) [p] / Anmashan (鞍馬山) [p] / 22.IX.2007, leg. M.-H. Tsou [p,w]” (TARI); 1♂: “TAIWAN: [p] / Kwantzeling, [p] / Tainan Hsien, 250m [p] / 6–7.IV.1965 [p, w] // C. Yoshimoto [p, w]” (BPBM); 2♂♂, 2♀♀: “Taiwan: Taipei (5934–5937) [p] / Chutzuhu (竹子湖) [p] / 15.VI.2008, leg. S.-F. Yu [p, w]” (TARI); 1♂: “Taiwan: Taipei (5661) [p] / Maokung (貓空) [p] / 29.V.2008, leg. S.-F. Yu [p, w]” (TARI); 1♂: “Taiwan: Taipei [p] / Pinglin (坪林) [p] / 28.VII.2007, leg. Y.-L. Lin [p, w]” (TARI); 1♀: “TAIWAN: [p] / Taipei & vicinity [p] / IX.1964 [p,w] // T. C. Maa [p] / Collector [p,w] // *Paridea* [h] / *angulicollis* [h] / 鑒定者: [p] Motschulsky [h, w]” (BPBM); 2♂♂, 2♀♀: “Taiwan: Taipei (916–919) [p] / Yangmingshan (陽明山) [p] / 10.III.2007, leg. M.-H. Tsou [p, w]” (TARI); 1♀: “TAIWAN, Ilan county, [p] / Mingchyh Forest [p] / Recreation Area, 1200 m, [p, w] // swept from vegetation, [p] / 5.IV.2002, [p] / leg. Gy. Fábián & O. Merkl [p, w]” (HNHM); 1♂: “TAIWAN bor. or. 2.vi.2008 [p] / Yilan County, 50 m [p] /cca 20 km N of Yilan City [p] / N 24°49,25´; E 121°44,39´ [p] / leg. F. & L. Kantner [p, w]” (FKCC). Each paratype has a type label: “**Paratypus** [p] / *Paridea (Semacia)* ♂ [or ♀] [p] / *kaoi* n. sp. [p] / des. C.-F. Lee, 2014 [p, pink label]”

#### Diagnosis.

This new species is similar to *Paridea angulicollis* but differs by the separation between basal black lateral margins and subapical spots on the elytra (Fig. 8) (connected in *Paridea angulicollis* (Fig. 98)), the wider apical processes of the eighth abdominal tergite in males (Fig. 26) (more slender in *Paridea angulicollis* (Fig. 107)), the wider penis in lateral view, and the deep notch on apical margin of the fifth abdominal ventrite in females (Fig. 25) (one pair of rounded processes in *Paridea angulicollis* (Fig. 106)).

**Figures 21–27.**
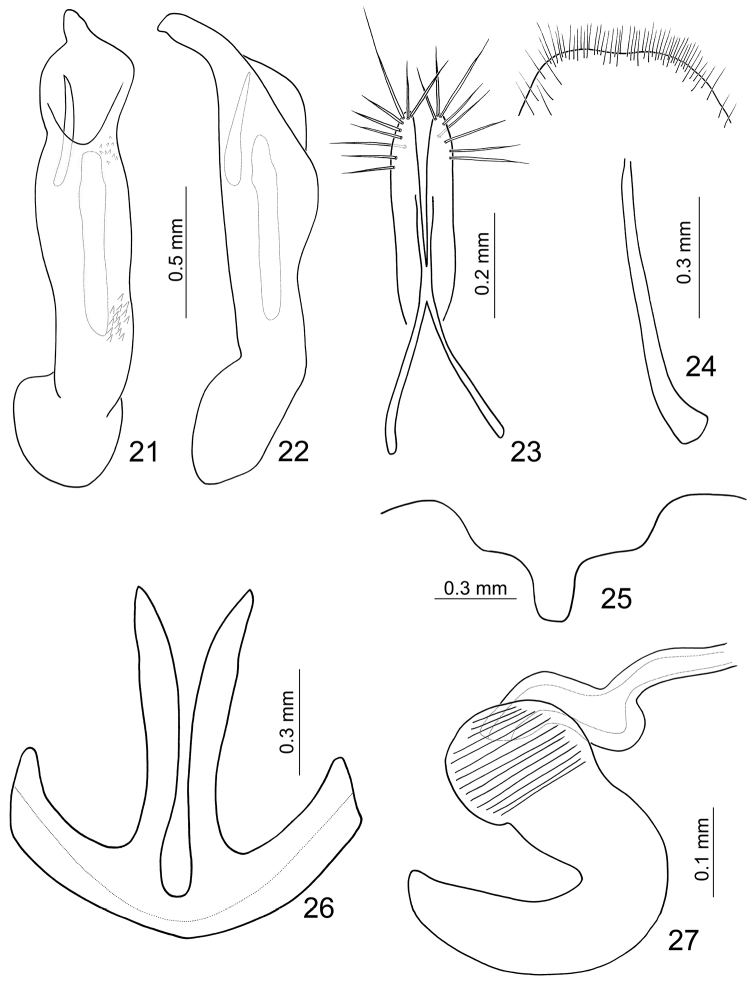
*Paridea (Semacia) kaoi* sp. n. **21** Penis, dorsal view **22** Penis, lateral view **23** Gonocoxae **24** Eighth abdominal sternite **25** Fifth abdominal ventrite **26** Eighth abdominal tergite **27** Spermatheca.

#### Males.

Length 5.0–5.3 mm, width 2.7–3.1 mm. Head and prothorax yellowish brown (Figs 6–8), labrum black, antenna brown; scutellum pale yellow; elytra pale yellow, postscutellar common area depressed; with one extremely slender black stripe along suture behind excavation, sometimes reduced; with one pair of large black spots subapically, lateral margin and epipleuron black, abbreviated at middle and separated from subapical black spots; meso- and metathoracic ventrites black; legs dark brown, apex of femur and base of tibia paler; abdomen yellow. Eighth abdominal tergite (Fig. 26) strongly sclerotized, transverse and slender, with one pair of slender and curved processes. Pygidium slightly projecting beyong elytral apices. Penis (Figs 21–22) strongly asymmetric, moderately narrowed at apical 1/6; apex narrow, tubular, and small; straight from lateral view; endophallic sclerites with one pointed sclerite, one elongate sclerite, an anterior cluster of small setae, and a posterior cluster of large setae.

#### Females.

Length 5.7–6.1 mm, width 3.3–3.5 mm. Color (Fig. 9) similar to male; elytra without excavation but with black spot instead. Apical margin of last abdominal ventrite (Fig. 25) with shallow emargination at middle, margin of emargination truncate with deep notch at middle. Pygidium slightly projecting beyong elytral apices. Gonocoxae (Fig. 23) slender, apex of each gonocoxa with eight or nine setae from apical 1/7 to apex; connection of gonocoxae extremely slender, base slender. Sternite VIII (Fig. 24) weakly sclerotized; apex narrow, apical margin a little emarginate at middle, surface with dense long setae along apical margin, spiculum short. Spermathecal receptaculum (Fig. 27) swollen; pump much longer than receptaculum, strongly curved; spermathecal duct short, stout, shallowly projecting into receptaculum.

#### Etymology.

The name is dedicated to Mr. Shu-Jung Kao who financially supported the Taiwan Chrysomelid Research Team.

#### Distribution.

Taiwan. This species occurs in central and northern Taiwan (Fig. 18).

#### Host plant.

Cucurbitaceae: *Gynostemma pentaphyllum* (Thunb.) Makino.

#### Notes.

This species was misidentified as *Paridea (Semacia) angulicollis* by Chûjô (1938, 1962, 1963).

### Paridea (Semacia) sexmaculata

(Laboissière, 1930)

http://species-id.net/wiki/Paridea_sexmaculata

Semacia sexmaculata Laboissière, 1930: 336; Chûjô 1935: 168.
Paraulaca (Paraulaca) semaculata : Chûjô 1962: 194; Chûjô 1965: 97.
Paridea (Paraulaca) sexmaculata : Kimoto 1966: 30; Kimoto 1969: 33; Kimoto 1989: 250.
Paridea (Semacia) sexmaculata : Yang 1991: 268.
Paridea (Paridea) sexmaculata : Beenen 2010: 468.
Paraulaca taiwana Chûjô, 1935: 167 (part).

#### Type locality.

Taiwan, Tainan.

#### Type material.

Holotype female (ZMUH), pinned, labeled: “Tainan [h] / Formose [h, w] // TYPE [red letters, p] / ♀ [h, w] // *Semacia* [h] / *sexmaculata* [h] / *m* [h] / V. Laboissière – Dét. [p, w] // Le Moult Vend. [p] / via Reinbek [p] / Eing Nr. 1, 1957 [p, w]”. It was indicated as male in the original description but marked as female in the examined specimen.

#### Additional material examined

**(n = 36).** **CHINA:** **Hunan:** 1♀, Yungshun, 6.VIII.1988, leg. S.-U. Wang (TARI); **TAIWAN: Hsinchu:** 1♂, Shinchiku (= Hsinchu), 1–30.VII.1918, leg. J. Sonan & K. Miyake (BMNH); 1♂,Wuchihshan, 27.III.2008, leg. S.-F. Yu (TARI); **Hualien:** 2♂♂, 1 ♀, YuShan N. P., 8.VI.2008, leg. F. & L. Kantner (FKCC, JBCB), 1 ♀, same locality, 7.VI.2008 (FKCC); **Ilan:** 1♂, Fushan Botanical Garden, 8.-11.IV.2002, leg. O. Merkl (HNHM); 1♀, Taipingshan, 12.VI.2007, leg. Y.-C. Chang (TARI); 1♀, Tulishan, 10.III.2007, leg. H.-H. Li (TARI); **Kaoshiung:** 1♀, Erchituan, 1.V.2009, leg. U. Ong (TARI); 1♂, Tona logging trail, 3.II.2013, leg. B.-X. Guo (TARI); 1♂, Wukungshan, 23.I.2009, leg. M.-H. Tsou (TARI); **Nantou:** 1♀, Tungpu, 28.IV-2.V.1981, leg. T. Lin & C. J. Lee; 1♀, 19–23.VII.1982, leg. leg. L. Y. Chou & T. Lin (TARI); **Pingtung:** 2♂♂, Machia, 11.III.2013, leg. Y.-T. Chung (TARI); 1♂, same locality, 17.III.2013, leg. W.-C. Liao (TARI); 1♀, Nanjenshan, 1.III.2010, M.-L. Jeng (TARI); 1♀, Peitawushan, 18.V I.2012, leg. J.-C. Chen (TARI); 1♂, Tahanshan, 18.VII.2007, leg. C.-F. Lee (TARI); 1♀, same locality, 27.VIII.2009, leg. J.-C. Chen (TARI); 1♀, same locality, 6.VI.2012, leg. C.-F. Lee (TARI); 1♂, same locality, 19.VII.2012, leg. C.-F. Lee (TARI); 1♂, same locality, 26.III.2013, leg. C.-F. Lee (TARI); **Taichung:** 1♀, Anmashan, 7.VI.2010, leg. C.-F. Lee (TARI); 1♂, Wushihkeng, 19.III.2008, leg. C.-F. Lee (TARI); **Taipei:** 1♂, Fushan, 12.III.2009, leg. H.-J. Chen (TARI); 1♀, Tunghou, 27.VI.2007, leg. M.-H. Tsou (TARI); 1♀, Wulai, 12.III.2009, leg. S.-F. Yu (TARI); **Taitung:** 1♀, Hsiangyang, 14.VIII.2012, leg. C.-F. Lee (TARI); 1♂, Liyuan, 19.VI.2013, leg. C.-F. Lee; 1♀, Motien, 23.VI.2010, leg. S.-F. Yu (TARI); 1♂, same locality, 19.VI.2011, leg. C.-F. Lee (TARI).

#### Diagnosis.

*Paridea (Semacia) sexmaculata* is similar to *Paridea (Semacia) kaoi* sp. n. and *Paridea (Semacia) angulicollis* with the depression on postscutellar common area of males but flat and replaced with a black spot in females. It differs by the presence of a black spot near the lateral margin instead of black stripe along lateral margin in *Paridea (Semacia) kaoi* sp. n. and *Paridea (Semacia) angulicollis*, the well sclerotized and extremely slender penis (Fig. 37) and processes on eighth abdominal tergite of males (Fig. 42), and the presence of two acute processes on the fifth abdominal ventrite in females (Fig. 41).

#### Males.

**Figures 28–36.**
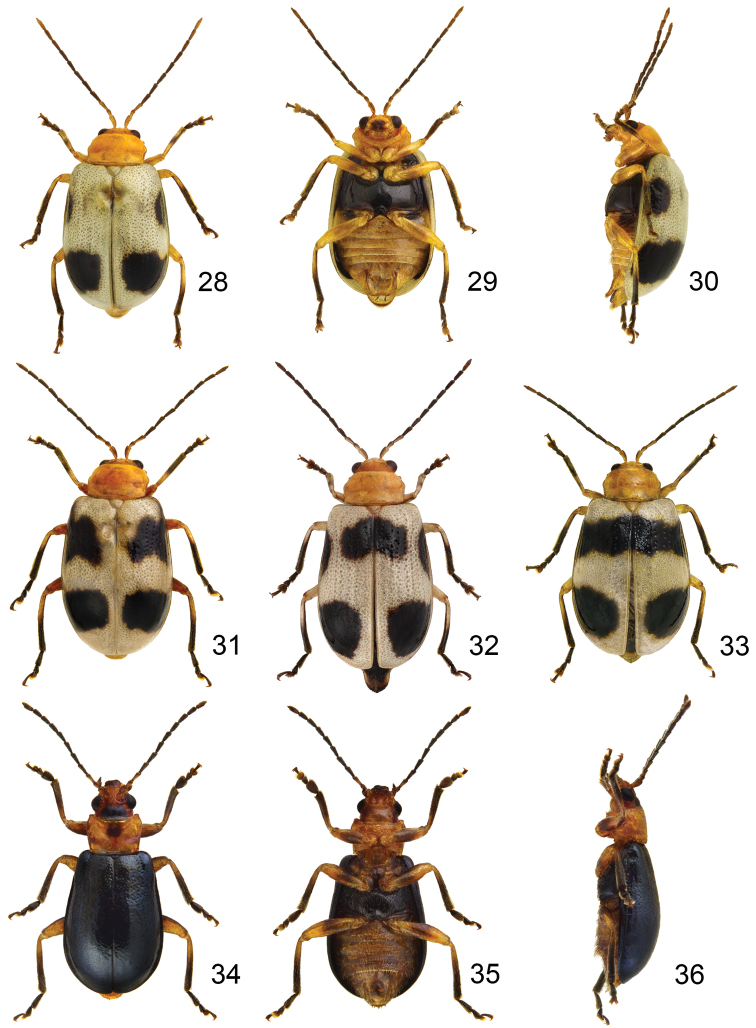
*Paridea* species. **28** *Paridea (Semacia) sexmaculata*, male, dorsal view **29** ditto, ventral view **30** ditto, lateral view **31** *Paridea (Semacia) sexmaculata*, male, color variation **32** *Paridea (Semacia) sexmaculata*, female, dorsal view **33** *Paridea (Semacia) sexmaculata*, female, color variation **34** *Paridea (Paridea) costata*, male, dorsal view **35** ditto, ventral view **36** ditto, ventral view.

**Figures 37–43.**
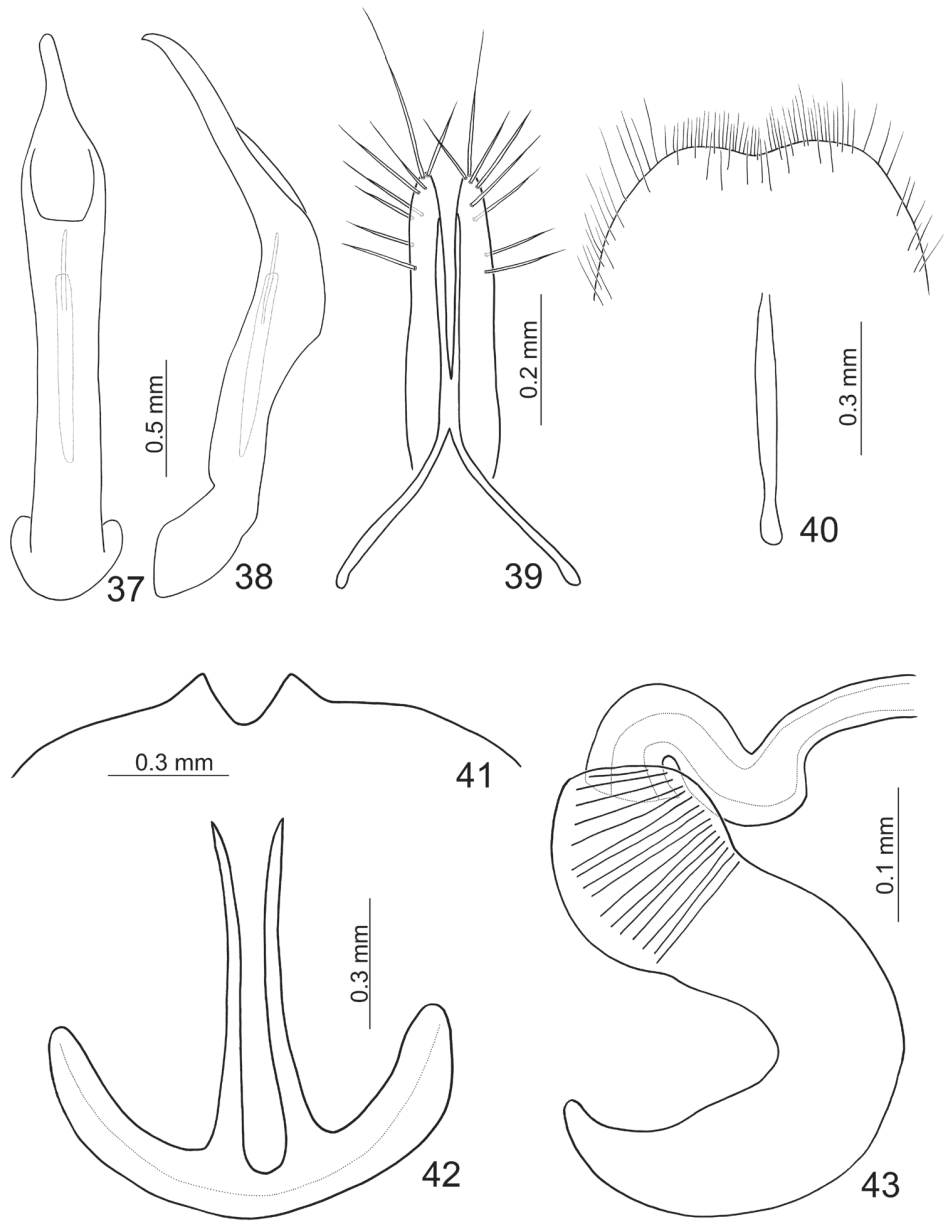
*Paridea (Semacia) sexmaculata*. **37** Penis, dorsal view **38** Penis, lateral view **39** Gonocoxae **40** Eighth abdominal sternite **41** Fifth abdominal ventrite **42** Eighth abdominal tergite **43** Spermatheca.

Length 5.1–6.0 mm, width 2.9–3.3 mm. Head and prothorax yellowish brown (Figs 28–30), labrum black, antenna blackish brown; scutellum pale yellow; elytra pale yellow, postscutellar common area depressed; with one pair of black spots near lateral margin at same level with excavation, spots extending inwards in some individuals (Fig. 31; one pair of large black spots subapically, lateral margin and epipleuron yellow; meso- and metathoracic ventrites black; legs yellowish brown, apical half of tibia, and tarsi black; abdomen yellow. Eighth abdominal tergite (Fig. 42) strongly sclerotized, transverse and slender, with one pair of extremely slender and curved processes. Pygidium slightly projecting beyong elytral apices. Penis (Figs 37–38) very slightly asymmetric, weakly narrowed at apical 1/6; apex narrow, tubular, and extremely long; moderately curved from lateral view; endophallic sclerites with one elongate sclerite and one layer of small setae.

#### Females.

Length 5.3–6.2 mm, width 3.1–3.4 mm. Similar to male; elytra without excavation but with transverse black spot instead (Fig. 32), sometimes connected with lateral black spots (Fig. 33). Apical margin of last abdominal ventrite (Fig. 41) with one pair of small, wide, and acute processes at middle, with shallow notch between processes. Pygidium slightly projecting beyong elytral apices. Gonocoxae (Fig. 39) slender, apex of each gonocoxa with eight setae from apical 1/4 to apex; connection of gonocoxae extremely slender, base slender. Sternite VIII (Fig. 40) weakly sclerotized; apex wide, apical margin slightly concave at middle, surface with dense long setae along apical margin, spiculum short. Spermathecal receptaculum (Fig. 43) slightly swollen; pump much longer than receptaculum, strongly curved; spermathecal duct short, stout, shallowly projecting into receptaculum.

#### Distribution.

Taiwan, China (Hunan). It is widespread in Taiwan (Fig. 19). Yang (1991) indicated that this species was also found in Hebei, Beijing, Jiangsu, Shanghai, Zheiang, Fujian, and Hainan provinces of China without voucher specimens. These records require confirmation.

#### Host plant.

Cucurbitaceae: *Gynostemma pentaphyllum* (Thunb.) Makino.

#### Notes.

Two specimens of the type series of *Paraulaca taiwana* were misidentified. They are *Paridea (Semacia) sexmaculata* and labeled as follow: 1♂: “Shiigo [= Maopu, Wufeng township] Chikuto [= Chutung] [p] / SHINCHIKU [= Hsinchu county] [p] / 27–30.VI.1934 [p] / COL. M. CHUJO [p, w] // COTYPE [p, circle label with yellow letters] // *Paraulaca* [h] / *taiwana* Chûjô [h] / DET. M. CHUJO [p, g] // No. 1339 [p, w]” (TARI); 1♂: “Formosa [p] / Karenko, [= Hualien] – 19. [p] / VII 20-VIII 4. [p] / T. Okuni, [p, w] // COTYPE [p, circle label with yellow letters] // *Paraulaca* [h] / *taiwana* Chûjô [h] / DET. M. CHUJO [p, g] // 2185 [p, w]” (TARI).

### Paridea (Paridea) costata

(Chûjô, 1935)

http://species-id.net/wiki/Paridea_costata

Paraulaca costata Chûjô, 1935: 164.
Paraulaca (Carapaula) costata : Chûjô 1962: 198 (redescription); Chûjô 1963: 395.
Paridea (Paridea) costata : Gressitt and Kimoto 1963: 512; Kimoto 1966: 29; Kimoto 1969: 33; Kimoto 1989a: 251; Kimoto 1991: 11; Takizawa et al. 1995: 12.

#### Type locality.

Taiwan, Hualien.

#### Type material.

Lectotype male (TARI), pinned, here designated to fix the concept of *Paraulaca costata* Chûjô and to ensure the universal and consistent interpretation of the same, labeled: “Formosa [p] / Karenko [= Hualien], -19 [p] / VII 20-VIII 4. T. Okuni, [p, w] / COTYPE [p, circle label with yellow letters] // *Paraulaca* [h] / *costata* [h] / Chûjô [h] / DET. M. CHUJO [p, g] // 1491 [p, w] // **Lectotypus** [p] / *Paraulaca costata* ♂ [p] / Chûjô, 1935 [p] / des. C.-F. Lee, 2014 [p, r]”. Paralectotypes: 1♂ (TARI): “Formosa [p] / Y. Miwa [p, w] // COTYPE [p, circle label with yellow letters] // *Paraulaca* [h] / *costata* [h] / Chûjô [h] / DET. M. CHUJO [p, g] // 2585 [p, w]”; 1♀ (TARI): “14/IV 1918 [h] / Bakuras [= Bakulasi, in Nantou] [h] / Col. I. Nitobe [p, w] // COTYPE [p, circle label with yellow letters] // *Paraulaca* [h] / *costata* [h] / Chûjô [h] / DET. M. CHUJO [p, g] // 1490 [p, w]”; 1♀ (TARI): “Horisha [= Puli, in Nantou] [h] / Apr 2. 1919 [h, w] // COTYPE [p, circle label with yellow letters] // *Paraulaca* [h] / *costata* [h] / Chûjô [h] / DET. M. CHUJO [p, g] // 2586 [p, w]”; 2♂♂ (SDEI): “Taihorin [= Talin, in Chiayi] [p] / Formosa [p] / H. Sauter, 1911 [p, w] // 7.VIII. or 7.VII. [p, w] // Syntypus [p, r] // *Paraulaca* [h] / *costata* [h] / Chûjô [h] / DET. M. CHUJO [p, g] // DEI Müncheberg [p] / Col – 04199 and 04200 [p, g]”. Each paralectotype has a type label: “**Paralectotypus** [p] / *Paraulaca costata* ♂ [or ♀] [p] / Chûjô, 1935 [p] / des. C.-F. Lee, 2014 [p, pink label]”

#### Additional material examined

**(n = 29).** **TAIWAN:** **Hualien:** 1♀, Taroko N. P., 3.vi.2008, F. & L. Kantner leg. (JBCB); **Hsinchu:** 1♀, Mamei, 4.V.2008, leg. S.-F. Yu (TARI); **Ilan:** 1♀, Suchi, 19.V.2010, leg. H.-J. Chen (TARI); **Nantou:** 3♂♂, 1♀, Sungkang, 2.VII.2008, leg. M.-H. Tsou (TARI); 2♂♂, 1♀, Tungpu, 28.IV.-2.V.1981, leg. T. Lin & C. J. Lee (TARI); 1♂, Wushe, 30.VIII–2.IX.1982, leg. L. Y. Chou & K. C. Chou (TARI); 1♂, same locality, 19–22.IV.1983, leg. K. C. Chou & S. P. Huang (TARI); **Pingtung:** 1♀, Suchunghsi, 8.V.2013, leg. Y.-T. Chung; **Taichung:** 2♂♂, 1♀, Kukuan, 16.VII.2007, leg. M.-H.Tsou (TARI); 1♀, Wuling, 30.VI.2008, leg. S.-F. Yu (TARI); **Taoyuan:** 1♀, Hsuanyuan, 16.III.2008, leg. S.-F. Yu (TARI); 6♂♂, 1♀, Tamanshan, 2.VIII.2008, leg. S.-F. Yu (TARI); 1♂, 5♀♀, same locality, 2.VIII.2008, leg. M.-H. Tsou (TARI).

#### Diagnosis.

*Paridea (Paridea) costata* is recognized by the black vertex, presence of one black spot on the pronotum, the metallic blue elytra, and the swollen first tarsomeres of front and middle legs of males.

#### Males.

**Figures 44–51.**
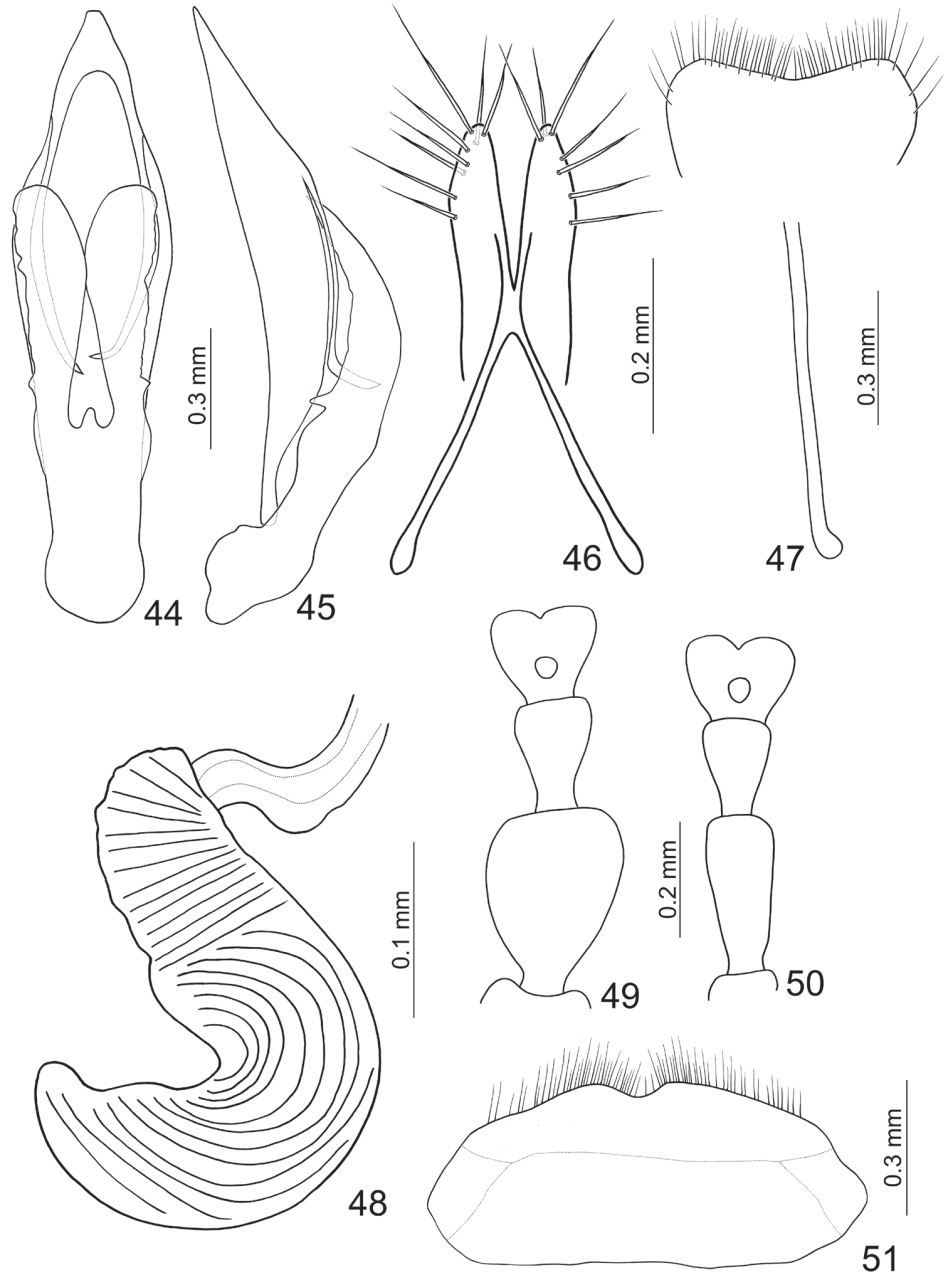
*Paridea (Paridea) costata*. **44** Penis, dorsal view **45** Penis, lateral view **46** Gonocoxae **47** Eighth abdominal sternite **48** Spermatheca **49** Tarsi of front leg, male **50** Tarsi of front leg, female **51** Eighth abdominal tergite.

Length 4.5–4.7 mm, width 2.2–2.4 mm. General color (Figs 34–36) yellowish brown; antenna blackish brown but three basal antennomeres paler; vertex with one big black spot; pronotum with a moderate black spot near center; scutellum and elytra bluish black; metathoracic ventrites black; tibia and tarsi dark brown; femora darkened except bases and apices. Elytra with one longitudinal costa arising from humerus, reduced in some individuals. Median lobe at fifth abdominal ventrite deeply depressed. Eighth abdominal tergite (Fig. 51) weakly sclerotized but apex well sclerotized, transverse and wide, apical margin emarginate at middle, with dense long setae along apical margin. First tarsomeres of pro- and mesotarsi swollen (Fig. 49). Penis (Figs 44–45) wide, apically tapering, apex truncate, slightly curved near based from lateral view; with one pair of elongate processes extending from near apex to middle, base curved upwards from lateral view; external process large and wide, lateral margin irregular from middle to apex, with a small process at middle of lateral margin; endophallus without visible sclerites.

#### Females.

Length 4.7–4.9 mm, width 2.5 mm. Similar to male; apical margin of last abdominal ventrite smooth, not modified. Gonocoxae (Fig. 46) slender, apex of each gonocoxa with seven or eight setae from apical 1/7 to apex; connection of gonocoxae extremely slender, base slender. Sternite VIII (Fig. 47) weakly sclerotized; apex wide, apical margin emarginate at middle, surface with dense long setae on apical margin, spiculum short. Spermatheca (Fig. 48) strongly sclerotized, disk with dense transverse grooves; receptaculum narrower than pump; pump short and wide, strongly curved; spermathecal duct short, stout, shallowly projecting into receptaculum.

#### Distribution.

China (Gansu, Jiangxi, Sichuan, Zhejiang), Taiwan. It is uncommon but widespread in Taiwan (Fig. 20).

#### Host plants.

Cucurbitaceae: *Thladiantha nudiflora* Hemsl. ex Forbes & Hemsl.

### Paridea (Paridea) cyanipennis

(Chûjô, 1935)

http://species-id.net/wiki/Paridea_cyanipennis

Paraulaca cyanipennis Chûjô, 1935: 164
Paraulaca (Paraulaca) cyanipennis : Chûjô 1962: 192 (redescription);
Paridea (Paridea) cyanipennis : Kimoto 1966: 30; Kimoto 1989a: 250; Kimoto 1991: 11.

#### Type locality.

Taiwan, Pingtung, Henchun (= Koshun), 22°00’N, 120°44E, 50 m, broad-leaf forest.

#### Type material.

Holotype male (SDEI), mounted on card, labeled: “ Kankau (Koshun) [p] / Formosa [p] / H. Sauter VI. 1912 [p, w] // Holotype [h, red letters] // *Paraulaca* [h] / *cyanipennis* [h] / Chûjô [h] / DET. M. CHUJO [p, g] // DEI Müncheberg [p] / Col – 04201 [p, g]”. Paratypes: 1♂ (SDEI), same as holotype but with “Paratype” and “Col. – 04204”; 1♂ (TARI), labeled: “ Kankau (Koshun) [p] / Formosa [p] / H. Sauter VII. 1912 [p, w] // Paratype [h, red letters] // *Paraulaca* [h] / *cyanipennis* [h] / Chûjô [h] / DET. M. CHUJO [p, g] // 2848 [p, w]”; 1♂, 2♀♀ (SDEI), labeled: “ Kankau (Koshun) [p] / Formosa [p] / H. Sauter IX. 1912 [p, w] // Paratype [red letters, h] // *Paraulaca* [h] / *cyanipennis* [h] / Chûjô [h] / DET. M. CHUJO [p, g] // DEI Müncheberg [p] / Col – 04202, 04205–6 [p, g]”; 1♂ (TARI), same as preceding but with “2847 [p, w]”; 1♀ (SDEI), same as preceding but with “Allotype” and “DEI Müncheberg [p] / Col – 04203 [p, g]”.

#### Additional material examined

**(n = 16).** **TAIWAN:** **Hualien:** 2♀♀, Wenlan, 21.X-9.XII.2009, leg. W. T. Yang & K. W Huang (NMNS); **Kaoshiung:** 1♂, Chiasien, 10–13.V.1981, leg. C. C. Chen & C. C. Pan (TARI); 1♂, 1♀, Meinung, 17.VII.2012, leg. J.-C. Chen (TARI); 3♂♂, 2♀♀, Tengchih, 26.IV.2010, leg. J.-C. Chen (TARI); **Pingtung:** 1♂, Checheng, 1.XII.2012, leg. J.-C. Cheng (TARI); 2♂♂, 2♀♀, Kueishan, 3.VIII.2012, leg. J.-C. Chen (TARI); 1♀, Lilungshan, 6.VI.2013, leg. J.-C. Chen (TARI).

#### Diagnosis.

This species is similar to *Paridea (Paridea) costata* with the metallic blue elytra but differs in possessing a yellowish brown lateral margin of each elytron, the yellowish brown head and pronotum, and the unmodified first tarsomeres of males.

#### Males.

**Figures 52–57.**
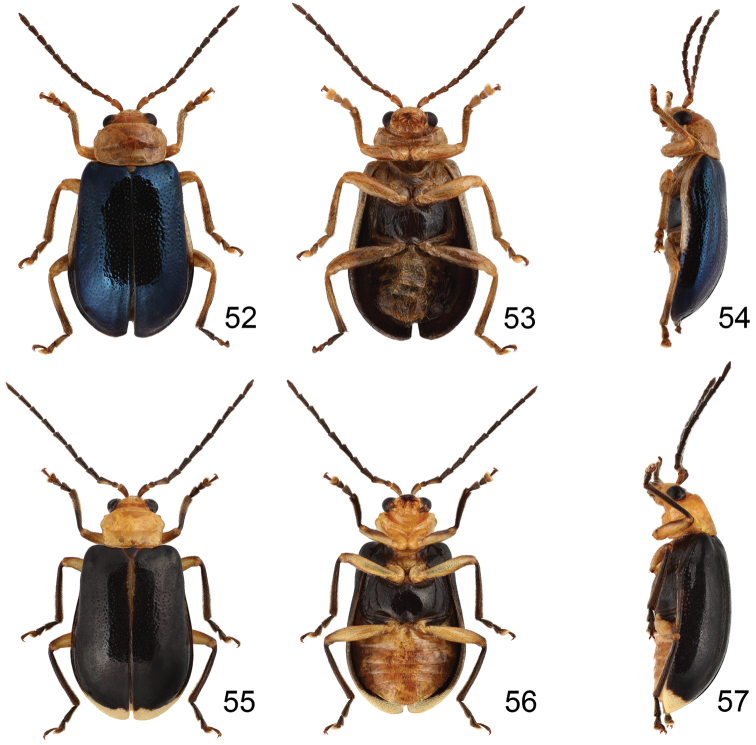
*Paridea* species. **52** *Paridea (Paridea) cyanipennis*, male, dorsal view **53** ditto, ventral view **54** ditto, ventral view **55** *Paridea (Paridea) sauteri*, male, dorsal view **56** ditto, ventral view **57** ditto, ventral view.

**Figures 58–63.**
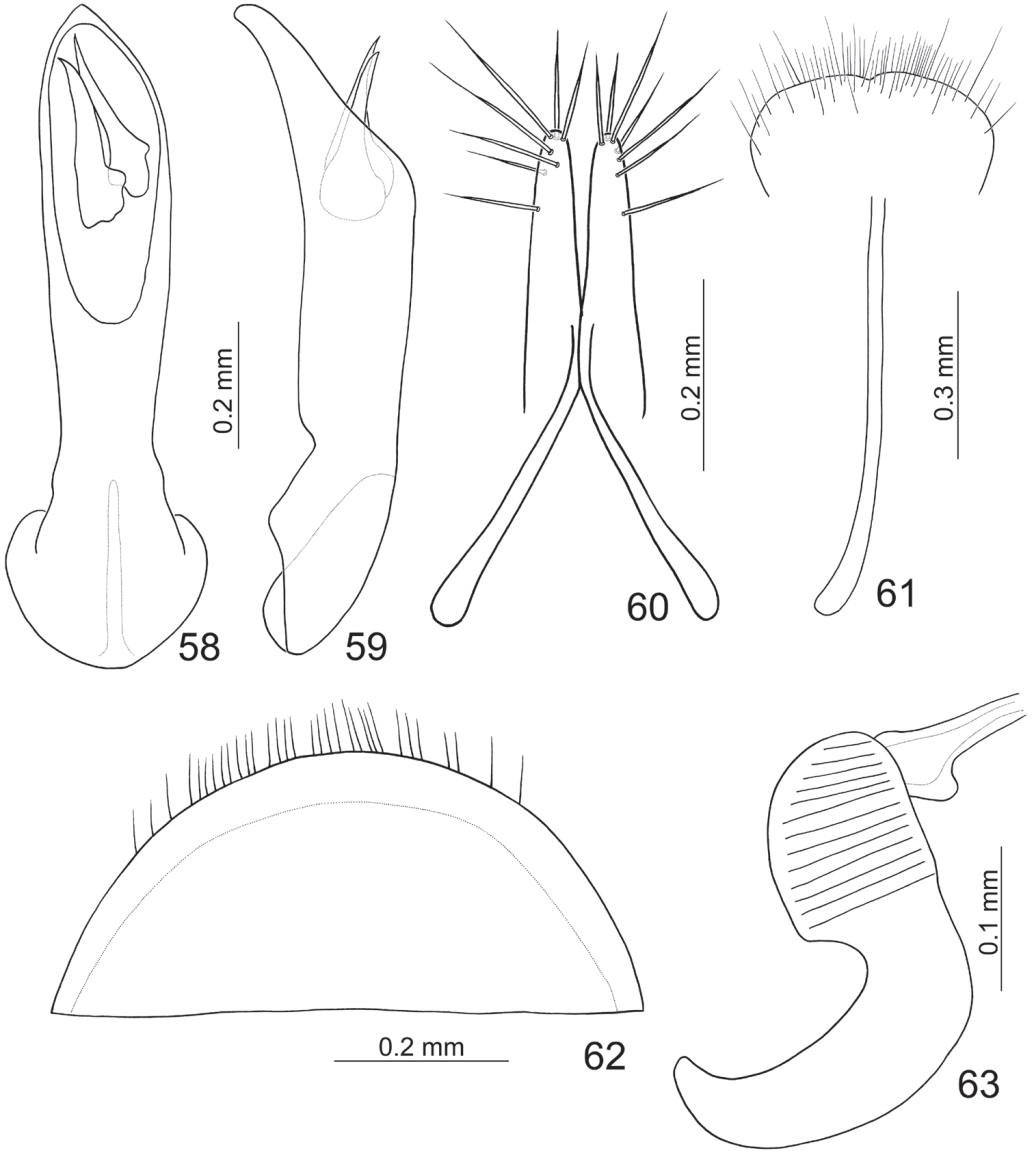
*Paridea (Paridea) cyanipennis*. **58** Penis, dorsal view **59** Penis, lateral view **60** Gonocoxae **61** Eighth abdominal sternite **62** Eighth abdominal tergite **63** Spermatheca.

Length 4.9 mm, width 2.4 mm. General color yellowish brown (Figs 52–54); antennomeres IV-XI darkened; elytra metallic blue, but basal margin, base to apical 1/3 of lateral margin yellowish brown; metathoracic ventrites metallic blue. Elytra with one longitudinal costa arising from humerus, reduced in some individuals. Eighth abdominal tergite (Fig. 62) weakly sclerotized but apex well sclerotized, transverse and wide, apical margin rounded, with dense long seta along apical margin. Penis (Figs 58–59) wide, apically tapering, apex slightly asymmetric and pointed, slightly curved near apex and apex narrowly rounded from lateral view; endophallus with two visible sclerites, apically curved and pointed, one smaller.

#### Females.

Length 5.5–6.1 mm, width 3.0–3.2 mm. Similar to male; apical margin of last abdominal ventrite smooth, not modified. Gonocoxae (Fig. 60) slender, apex of each gonocoxa with seven setae from apical 1/7 to apex; connection of gonocoxae extremely slender, base slender. Sternite VIII (Fig. 61) weakly sclerotized; apex wide, apical margin emarginate at middle, surface with longer setae near apical margin and shorter setae on apical margin, spiculum long. Spermatheca (Fig. 63) weakly sclerotized; receptaculum slightly swollen; pump short and wide, strongly curved; spermathecal duct short, stout, shallowly projecting into receptaculum.

#### Distribution.

**Figures 64–67.**
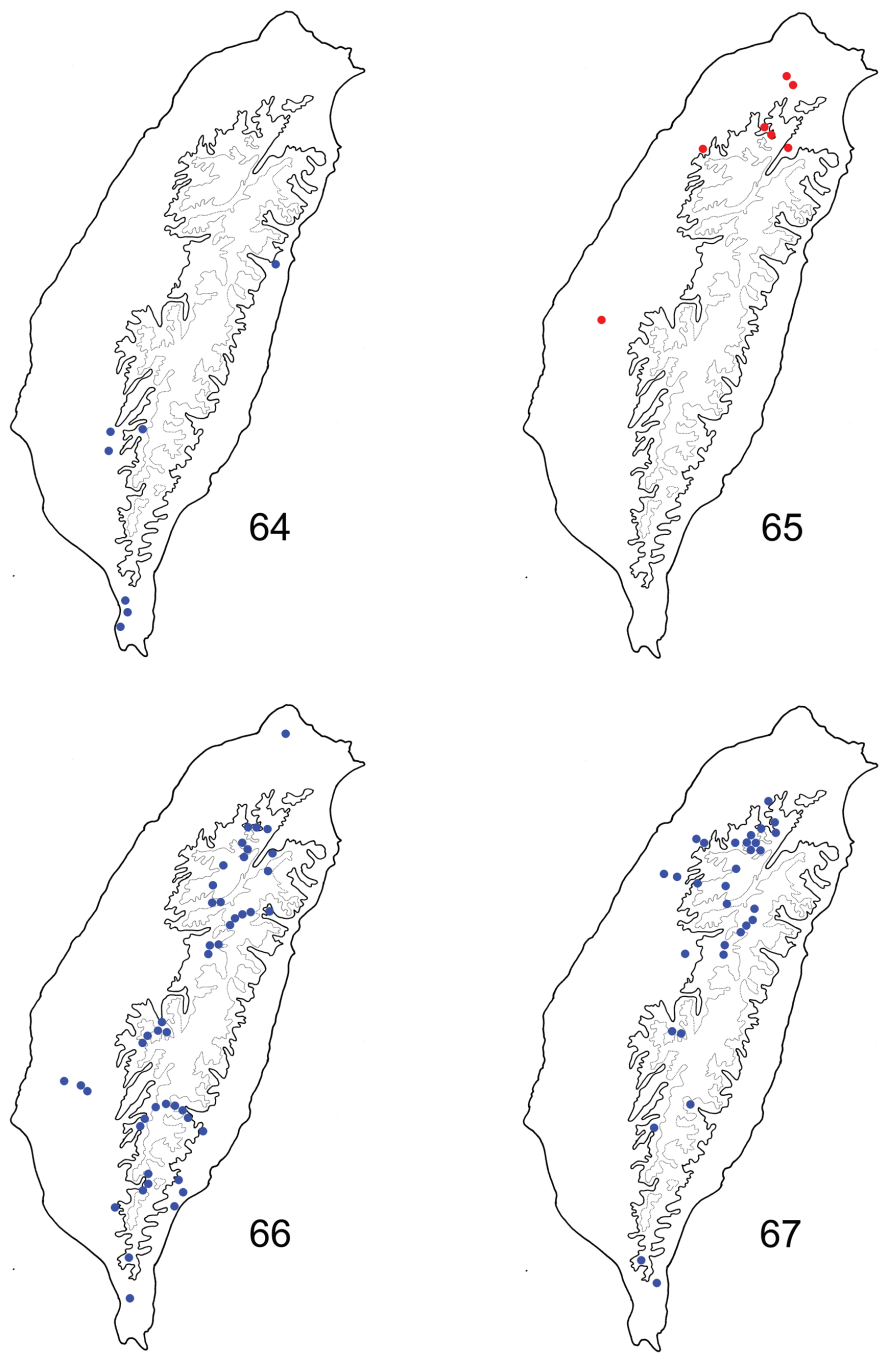
Distribution map of *Paridea* species, solid line: 1000 m, broken line: 2000 m. **64** *Paridea (Paridea) cyanipennis* **65** *Paridea (Paridea) sauteri* **66** *Paridea (Paridea) taiwana* **67** *Paridea (Paridea) testacea*.

The species occurs in eastern and southern Taiwan (Fig. 64).

#### Host plant.

Cucurbitaceae: *Momordica cochinchinensis* (Lour.) Spreng.

### Paridea (Paridea) sauteri

(Chûjô, 1935) stat. r.

Paraulaca sauteri Chûjô, 1935: 166; Kimoto 1974: 26 (as synonym of *Paridea sinensis*)
Paraulaca (Paraulaca) sauteri : Chûjô 1962: 192 (redescription); Chûjô 1963: 395.
Paridea (Paraulaca) sauteri : Kimoto 1965a: 489.
Paridea (Paridea) sauteri : Kimoto 1966: 29; Kimoto 1969: 35; Takizawa et al. 1995: 12.
Paridea (Paridea) sinensis : Kimoto 1986: 57; Kimoto 1989a: 251; Kimoto 1991: 11.

#### Type locality.

Taiwan, Chiayi, Talin (= Taihorin), 23°35’N, 120°28’E, 50 m, broad-leaf forest.

#### Type material.

Holotype ♂ (SDEI), labeled: “Taihorin [p] / Formosa [p] / H. Sauter, 1911 [p] // 7. VIII. [p] // Holotype [h, red letters] // *Paraulaca* [h] / *sauteri* [h] / Chûjô [h] / DET. M. CHUJO [p, g] // DEI Müncheberg [p] / Col – 04207 [p, g]”. Paratypes: 1♀ (SDEI), same as holotype but with “Allotype” and “Col – 04210”; 2♀♀ (SDEI), labeled: Taihorin [p] / Formosa [p] / H. Sauter, 1911 [p] // 7. VII. [p] // Paratype [h, red letters] // *Paraulaca* [h] / *sauteri* [h] / Chûjô [h] / DET. M. CHUJO [p, g] // DEI Müncheberg [p] / Col – 04208 & 04209 [p, g]”; 1♀ (TARI), same as preceding, but with “2846 [p, w]”; 1♂ (TARI), labeled: “Taihorin [h] / Formosa [p] 10. [h] / Sauter [p] IV. [h] __ 07 09 // Paratype [h, red letters] // *Paraulaca* [h] / *sauteri* [h] / Chûjô [h] / DET. M. CHUJO [p, g] // 2845 [p, w]”.

#### Additional material examined

**(n = 51).** **TAIWAN:** **Hsinchu:** 13♂♂, 3♀♀, Wufeng, 14–16.VII.1982, leg. **K. C. Chou & C. C. Pan (TARI)**; Ilan: **2**♂♂, Songluo, 7.IV.2007, leg. M.-H. Tsou (TARI); **Taipei:** 2♂♂, Fushan, 5.IV.2007, leg. S.-F. Yu (TARI); 3♂♂, same locality, 2.III.2012, leg. M.-H. Tsou (TARI); 1♂, Shintien, 2.X.2010, leg. Y.-F Hsu (TARI); 1♂, Wulai, 7.X.2006, leg. S.-F. Yu (TARI); 1♂, same locality, 30.III.2007, leg. C.-F. Lee (TARI); 1♀, same locality, 16.V.2007, leg. G. Martin & J. Quicke (BMNH); 2♂♂, same locality, 15.VI.2007, leg. C.-F. Lee (TARI); 8♂♂, same locality, 26.VI.2009, leg. C.-F. Lee (TARI); 2♂♂, same locality, 26.VII.2009, leg. H.-J. Chen (TARI); 7♂♂, same locality, 30.VIII.2009, leg. C.-F. Lee (TARI); 1♂, same locality, 1.III.2010, leg. C.-F. Lee (TARI); **Taoyuan:** 1♂, Hsiaowulai, 19.IV.2008, leg. S.-F. Yu (TARI); 1♂, Paling, 2.IX.2009, leg. H. Lee (TARI).

#### Diagnosis.

*Paridea (Paridea) sauteri* is similar to some individuals of *Paridea (Paridea) taiwana* having yellow apices of the black elytra but differs by possessing yellow femora and black tibiae (black outer margins of femora and tibiae in *Paridea (Paridea) taiwana*).

#### Males.

**Figures 68–73.**
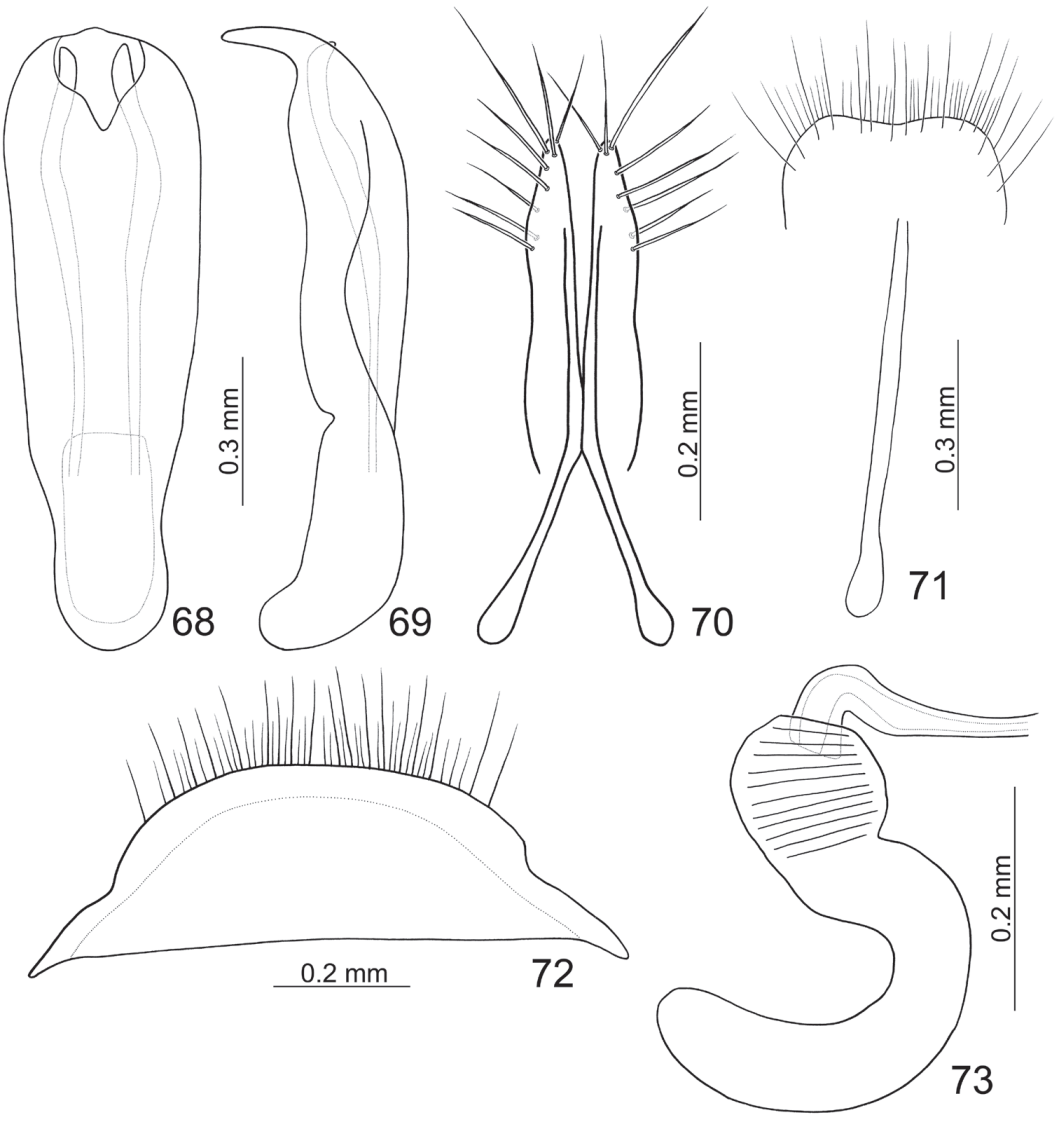
*Paridea (Paridea) sauteri*. **68** Penis, dorsal view **69** Penis, lateral view **70** Gonocoxae **71** Eighth abdominal sternite **22** Eighth abdominal tergite **73** Spermatheca.

Length 4.8–5.5 mm, width 2.9–3.2 mm. General color (Figs 55–57) yellowish brown; antenna dark brown; apex of labrum darkened; elytra black but apex pale yellow; scutellum, meso- and metachoracic ventrites black; tibiae and tarsi blackish brown. Eighth abdominal tergite (Fig. 72) weakly sclerotized, transverse and wide, apical margin truncate, with dense long seta along apical margin. Penis (Figs 68–69) wide, apex pointed, ventrally curved; almost straight from lateral view; endophallic sclerites composed of one pair of elongate and longitudinal sclerites, curved inwards near apex, apex projecting from opening.

#### Females.

Length 5.5–5.7 mm, width 3.2–3.3 mm. Similar to male; apical margin of last abdominal ventrite smooth, not modified. Gonocoxae (Fig. 70) slender, apex of each gonocoxa with eight setae from apical 1/4 to apex; connection of gonocoxae extremely slender, base slender. Sternite VIII (Fig. 71) weakly sclerotized; apex wide, apical margin slightly emarginate at middle, surface with longer setae near apical margin, and shorter and denser setae on apical margin, spiculum long. Spermathecal receptaculum (Fig. 73) slightly swollen; pump extremely long, strongly curved; spermathecal duct short, stout, shallowly projecting into receptaculum.

#### Distribution.

Endemic to Taiwan, and with a scattered distribution. The species seems to be allopatric with *Paridea (Paridea) taiwana* (Fig. 65). For example, it is common in Wulai, northern Taiwan. But no individuals of *Paridea (Paridea) taiwana* have been found in Wulai.

#### Host plants.

Cucurbitaceae: *Thladiantha nudiflora* Hemsl. ex Forbes & Hemsl.

### Paridea (Paridea) taiwana

(Chûjô, 1935) stat. r.

Paraulaca taiwana Chûjô, 1935: 167; Kimoto 1966: 29 (as synonym of *Paridea sauteri*).
Paraulaca (Carapaula) taiwana : Chûjô 1962: 200 (redescription); Chûjô 1965: 98.

#### Type locality.

Taiwan, Hualien.

#### Type material.

Lectotype male (TARI), pinned, here designated to fix the concept of *Paraulaca taiwana* Chûjô and to ensure the universal and consistent interpretation of the same, labeled: “Formosa [p] / Karenko [= Hualien], -- 9. [p] / VII 20-VIII 4. [p] / T. Okuni, [p, w] // COTYPE [p, circle label with yellow letters] // *Paraulaca* [h] / *taiwana* Chûjô [h] / DET. M. CHUJO [p, g] // 2186 [p, w] // **Lectotypus** [p] / *Paraulaca taiwana* ♂ [p] / Chûjô, 1935 [p] / des. C.-F. Lee, 2014 [p, r]”. Paralectotypes: 3♀♀ (TARI), same as lectotype but with “756, 2188, and 2189”; 1♂ (TARI): “Arisan [= Alishan], [h] / 1912-X-10 [h] / Col. I. Nitobe [p, w] // *Paridea* n sp. [h] / Det. Shiraki [p, w] // *Aulacophora* ? [h] / *quadriplagiata* Baly [h] / Det. Shiraki [p, w] // COTYPE [p, circle label with yellow letters] // *Paraulaca* [h] / *taiwana* Chûjô [h] / DET. M. CHUJO [p, g] // 755 [p, w]”; 1♀ (TARI): “Arisan [= Alishan] [p] / Formosa [p] / 25.X.1933 [p] / Col. M. CHUJO [p] // COTYPE [p, circle label with yellow letters] // *Paraulaca* [h] / *taiwana* Chûjô [h] / DET. M. CHUJO [p, g] // No. 1338 [p, w]”; 1♂ (TARI): “Rakuraku [in Hualien] [p] / 18.IV.1924 [p] / T. Shiraki [p, w] // COTYPE [p, circle label with yellow letters] // *Paraulaca* [h] / *taiwana* Chûjô [h] / DET. M. CHUJO [p, g] // 2191 [p, w]”; 1♀ (TARI): “Funkiko [= Fenchihu, in Chiayi] [h] / 16.II.1926 [h] / Col. J. Sonan [p, w] // COTYPE [p, circle label with yellow letters] // *Paraulaca* [h] / *taiwana* Chûjô [h] / DET. M. CHUJO [p, g] // 2190 [p, w]”; 1♀ (TARI): “Raisha [= Laiyi, in Pingtung] [p] / 30-VIII-1927 [p] / J. Sonan [p, w] // COTYPE [p, circle label with yellow letters] // *Paraulaca* [h] / *taiwana* Chûjô [h] / DET. M. CHUJO [p, g] // 2187 [p, w]”; 1♀ (SDEI): “Suisharyo [= Shuisheliao, in Chiayi] [p] / Formosa [p] / H. Sauter X.11 [p, w] // Syntypus [p, r] // *Paraulaca* [h] / *taiwana* [h] / Chûjô [h] / DET. M. CHUJO [p, g] // DEI Müncheberg [p] / Col – 04211 [p, g]”; 1♂ (SDEI): “Taihorinsho [= Talin, in Chiayi] [p] / Formosa [p] / H. Sauter [p] X 09 [h, w] // Syntypus [p, r] // *Paraulaca* [h] / *taiwana* [h] / Chûjô [h] / DET. M. CHUJO [p, g] // DEI Müncheberg [p] / Col – 04212 [p, g]”. Each paralectotype has a type label: “**Paralectotypus** [p] / *Paraulaca taiwana* ♂ [or ♀] [p] / Chûjô, 1935 [p] / des. C.-F. Lee, 2014 [p, pink label]”

#### Additional material examined

**(n = 503).** **TAIWAN:** **Chiayi:** 1♀, Arisan (= Alishan), 2–23.X.1918, leg. J. Sonan & M. Yoshino (BMHH); 2♀♀, Alishan, 5–9.VIII.1981, leg. L. Y. Chou and S. C. Lin; 1♀, same locality, 27.VI.2010, leg. U. Ong; 2♂♂, Laichitashan, 19.III.2009, leg. H. Lee; **Hsinchu:** 1♀, Litungshan, 3.V.2008, leg. Y.-L. Lin; 1♂, Mamei, 4.V.2008, leg. H.-F. Yu; 1♀, same locality, 18.V.2008, leg. M.-H. Tsou; 1♂, Shihlei, 6.III.2010, leg. Y.-L. Lin; 1♂, Talu logging trail, 29.IV.2008, leg. Y.-L. Lin; 1♀, same locality, 19.VI.2010, leg. Y.-L. Lin; 1♂, same locality, 17.III.2012, leg. Y.-L. Lin; **Hualien:** 1♂, Huitouwan, 10.VII.2007, leg. C.-F. Lee; 1♀, Tayuling, 9–16.VI.1980, leg. K. S. Lin and B. H. Chen; **Ilan:** 1♂, Mingchi, 20.IV.2007, leg. H.-H. Li; 1♀, Taipingshan, 12.VI.2007, leg. Y.-C. Chang; 1♂, Tuchang, 1.III.2007, leg. H.-H. Li; **Kaoshiung:** 1♂, Chungchihkuang, 16.IV.2012, leg. L.-P. Hsu; 2♂♂, 1♀, 10–13.X.2012, leg. L.-P. Hsu; 15♂♂, Erchituan, 1.III.2009, leg. U. Ong; 2♂♂, 1♀, same locality, 1.V.2009, leg. U. Ong; 3♂♂, 1♀, same locality, 21.VI.2010, leg. U. Ong; 1♂, same locality, 8.III.2013, leg. B.-X. Guo; 1♂, Southern C.-I. Hwy, 19.IX.2008, leg. L. Dembick (BMNH); 1♂, Shihshan logging trail, 23.III.2009, leg. H. Lee; 4♀♀, Tengchih, 2–5.VI.2008, leg. C.-F. Lee; 2♂♂, same locality, 12.III.2013, leg. Y.-T. Chung; 1♂, same locality, 19.III.2013, leg. Y.-T. Chung; 3♂♂, same locality, 31.III.2013, leg. W.-C. Liao; 2♂♂, same locality, 8.VI.2013, leg. W.-C. Liao; 1♀, same locality, 15.VI.2013, leg. B.-X. Guo; 2♂♂, same locality, 6.VII.2013, leg. W.-C. Liao; 2♂♂, 10.VIII.2013, leg. W.-C. Laio; 1♂, 1♀, Tona logging trail, 25.II.2013, leg. Y.-T. Chung; **Nantou:** 2♀♀, Hoshe, 22.VII.1982, leg. L. Y. Chou and T. Lin; 1♀, Hsitou, 12.IX.2009, leg. C.-F. Lee; 1♂, same locality, 10.V.2010, leg. Y.-T. Wang; 1♀, Lushan, 28.VI.1981, leg. K. S. Lin and W. S. Tang; 1♀, same locality, 6.VIII.2008, leg. H. Medel & M. V. L. Barclay (BMNH); 1♀, same locality, 8.VIII.2008, leg. H. Medel, U. Ong, M. V. L. Barclay & R. Ewers (BMNH); 1♀, Meifeng, 10.V.1979, leg. K. C. Chou; 3♂♂, 3♀♀, same locality, 20–22.VI.1979, leg. K. S. Lin and B. H. Chen; 2♀♀, same locality, 29.VIII-10.IX.1979; 3♂♂, same locality, 2–12.X.1979; 1♂, 1♀, same locality, 24.X.1979, leg. K. C. Chou; 1♂, 1♀, same locality, 25.X-7.XI.1979; 1♀, same locality, 2–4.VI.1980, leg. L. Y. Chou & C. C. Chen; 3♀♀, same locality, 8.VI.1980, leg. K. S. Lin and B. H. Chen; 1♂, 3♀♀, same locality, 26.VIII.1980, leg. K. S. Lin and C. H. Wang; 1♂, 4♀♀, same locality, 5–9.X.1980, leg. C. C. Chen and C. C. Chien; 4♂♂, 7♀♀, same locality, 7–9.V.1981, leg. K. S. Lin and S. C. Lin; 2♀♀, same locality, 24–26.VI.1981, leg. K. S. Lin and W. S. Tang; 1♂, same locality, 28–29.VIII.1981, leg. L. Y. Chou and S. C. Lin; 1♀, same locality, 22.V.1982, leg. L. Y. Chou; 1♀, same locality, 15.VII.1982, leg. S. C. Lin and C. N. Lin; 1♂, same locality, 4–7.X.1982, leg. K. C. Chou; 1♂, 1♀, same locality, 8–11.V.1984, leg. K. C. Chou and C. C. Pan; 1♀, Mong Gwu, 14 km E of Puli, 20.IV.2002, leg. D. Anstine, G. Fabián & O. Merkl (JBCB); 1♀, Sungkang, 13–15.IX.1984, leg. K. S. Lin and S. C. Lin; 1♀, same locality, 3.VII.2008, leg. M.-H. Tsou; 1♀, Tsuifeng, 8.V.1981, leg. K. S. Lin and S. C. Lin; 1♀, same locality, 12–14.IX.1984, leg. K. S. Lin and S. C. Lin; 1♂, Tungpu, 20–22.VI.1980, leg. C. C. Chen; 6♂♂, 8♀♀, same locality, 25–29.IX.1980, leg. L. Y. Chou and T. Lin; 8♂♂, 9♀♀, same locality, 28.IV-2.V.1981, leg. T. Lin and C. J. Lee; 9♂♂, 6♀♀, same locality, 5–8.X.1981, leg. T. Lin and W. S. Tang; 9♂♂, 11♀♀, same locality, 18–23.XI.1981, leg. T. Lin and W. S. Tang; 9♂♂, 21♀♀, same locality, 19–23.VII.1982, leg. L. Y. Chou and T. Lin; 2♂♂, 4♀♀, same locality, 22–25.XI.1982, leg. K. C. Chou and S. P. Huang; 5♂♂, 6♀♀, same locality, 20–24.VI.1983, leg. K. C. Chou and C. Y. Wong; 16♂♂, 28♀♀, same locality, 23–27.VII.1984, leg. K. C. Chou and C. H. Yang; 2♂♂, same locality, XI.1985, leg. K. S. Lin; 1♂, 1♀, Salihsien-shi near Tungpu, 23.XI.2002, leg. L. Ronkay & O. Merkl (JBCB, HNHM); 2♂♂, 2♀♀, Wanfengtsun, 2.IV.2008, leg. W.-T. Liu; 1♀, same locality, 24.IV.2008, leg. W.-T. Liu; 1♂, same locality, 8.VII.2008, leg. W.-T. Liu; 2♂♂, Wushe, 19–23.VI.1979, leg. K. S. Lin and B. H. Chen; 1♀, same locality, 20–22.VI.1980, leg. C. C. Chen; 2♀♀, same locality, 8.X.1980, leg. C. C. Chen and C. C. Chien; 2♀♀, same locality, 6–11.V.1981, leg. K. S. Lin and S. C. Lin; 1♀, same locality, 23–28.VI.1981, leg. K. S. Lin and W. S. Tang; 1♀, same locality, 4.VIII.1981, leg. T. Lin and W. S. Tang; 1♂, same locality, 14.VII.1982, leg. S. C. Lin and C. N. Lin; 10♂♂, 4♀♀, same locality, 30.VIII.-2.IX.1982, leg. L. Y. Chou and K. C. Chou; 4♂♂, 1♀, same locality, 7–8.X.1982, leg. K. C. Chou; 3♂♂, 3♀♀, same locality, 19–22.IV.1983, leg. K. C. Chou and S. P. Huang; 2♂♂, 2♀♀, same locality, 7.V.1984, leg. K. C. Chou and C. C. Pan; 2♂♂, 2♀♀, same locality, 17.VIII.1984, leg. K. S. Lin; 1♂, same locality, 11–15.IX.1984, leg. K. S. Lin; 6♂♂, 1♀, same locality, 23.III.2009, leg. U. Ong; 1♂, same locality, 21.VI.2009, leg. U. Ong; 1♂, 11♀♀, Kao-Leng Dyi, 18 km W of Wushe, 18.–19.IV.2002, leg. D. Anstine, G. Fabián & O. Merkl (HNHM, 1♀ JBCB); **Pingtung:** 1♀, Lilungshan, 5.XI.2009, leg. M.-H. Tsou; 4♂♂, Machia, 11.III.2013, leg. Y.-T. Chung; 1♂, Peitawushan, 25.VI.2012, leg. J.-C. Chen; 2♂♂, 1♀, Tahanshan, 24.VI.2007, leg. C.-F. Lee; 4♂♂, 4♀♀, same locality, 18–20.VII.2007, leg. C.-F. Lee and M.-H. Tsou; 1♀, same locality, 25.V.2008, leg. C.-F. Lee; 1♂, 1♀, same locality, 4.VII.2008, leg. M.-H. Tsou; 2♀♀, 21.III.2009, leg. M.-H. Tsou; 1♀, same locality, 5.IV.2009, leg. C.-F. Lee; 1♀, same locality, 18.V.2009, leg. M.-L. Jeng; 1♂, same locality, 28.VI.2009, leg. Y.-T. Chung; 1♂, same locality, 7.IX.2009, leg. U. Ong; 1♀, same locality, 3.XI.2009, leg. M.-H. Tsou; 1♂, 11.I.2010, leg. J.-C. Chen; 1♂, same locality, 28.VIII.2010, leg. Y.-L. Lin; 1♀, same locality, 5.VII.2011, leg. M.-H. Tsou; 1♂, same locality, 14.VIII.2011, leg. Y.-T. Wang; 2♂♂, same locality, 6.I.2012, leg. Y.-T. Chung; 1♀, same locality, 3.VI.2012, leg. W.-C. Liao; 1♀, same locality, 30.VII.2012, leg. Y.-T. Chung; 3♂♂, same locality, 20.X.2012, leg. W.-C. Liao; 1♀, same localtity, 10.XI.2012, leg. W.-C. Liao; 1♂, same locality, 15.XII.2012, leg. W.-C. Liao; 1♂, same locality, 14.I.2013, leg. Y.-T. Chung; 3♂♂, same locality, 26.II.2013, leg. Y.-T. Chung; 1♀, same locality, 14.III.2013, leg. Y.-T. Chung; 8♂♂, 2♀♀, same locality, 26.III.2013, leg. C.-F. Lee; 1♂, 2♀♀, same locality, 3.IV.2013, leg. Y.-T. Chung; 2♀♀, same locality, 25.V.2013, leg. Y.-T. Chung; 1♀, same locality, 2.VII.2013, leg. Y.-T. Chung; 1♀, same locality, 3.VII.2013, leg. B.-X. Guo; 2♂♂, 1♀, same locality, 1.X.2013, leg. Y.-T. Chung; 6♂♂, 5♀♀, Wutai, 12.IV.2009, leg. U. Ong; 2♀♀, same locality 9–12.V.1009, leg. U. Ong; 1♂, same locality, 17.V.2009, leg. U. Ong; 1♂, same locality, 1.IV.2010, leg. U. Ong; **Taichung:** 2♂♂, Anmashan, 16.VII.2007, leg. M.-H. Tsou; 1♀, same locality, 22.IX.2007, leg. M.-H. Tsou; 2♀♀, same locality, 7.VI.2010, leg. C.-F. Lee; 2♂♂, Chiapaotai, 14–18.X.1980, leg. K. S. Lin and C. H. Wang; 2♀♀, Kukuan, 14–17.X.1980, leg. K. S. Lin and C. H. Wang; **Tainan:** 2♂♂, 1♀, Kantoushan, 14.III.2010, leg. M.-H. Tsou; 1♀, Meiling, 28.XII.2008, leg. U. Ong; 2♂♂, 5♀♀, same locality, 24.III.2011, leg. U. Ong; 1♀, same locality, 24.IV.2013, leg. B.-X. Guo; 1♂, same locality, 7.VI.2013, leg. Y.-T. Chung; 1♂, Pichien, 11.II.2009, leg. U. Ong; **Taipei**, 1♀, Chutzuhu, 26.V.1983, leg. K. C. Chou; 1♂, same locality, 29.IV.2007, leg. M.-H. Tsou; **Taitung:** 1♀, Chihpen, 17–18.II.1982, leg. L. Y. Chou and K. C. Chou; 2♂♂, 1♀, Guanshan, 31.X.2009, leg. P.-F. Wang; 7♂♂, Hsiangyang, 14.VIII.2012, leg. C.-F. Lee; 1♂, Lichia, 2.VI.2009, leg. U. Ong; 1♀, Litao, 23.VI.2010, leg. M.-H. Tsou; 1♀, same locality, 4.X.2010, leg. M.-H. Tsou; 1♀, Liyuan, 19.VI.2013, leg. C.-F. Lee; 5♀♀, Motien, 23–24.VI.2010, leg. M.-H. Tsou; 12♂♂, same locality, 5.X.2010, leg. C.-F. Lee; 1♂, same locality, 19.VI.2011, leg. C.-F. Lee; 4♂♂, Taimali, 20.III.2008, P.-F. Wang; 1♂, Wulu logging trail, 26.IX.2007, leg. J.-F. Tsai; **Taoyuan:** 1♂, Paling, 3–5.V.1983, leg. K. C. Chou and C. C. Pan; 2♂♂, 2♀♀, Sankuang, 17.X.2009, leg. Y.-L. Lin. If not otherwise stated, all specimens deposited in TARI.

#### Diagnosis.

This species is similar to *Paridea (Paridea) sinensis* in general color pattern but differs in possessing extremely variable black spots on the elytra, the smaller external process on the penis and the abruptly widened apex of the penis in lateral view (the external processes are extremely long and the penis is slender in lateral view in *Paridea (Paridea) sinensis*) and the modified apical margin of the fifth abdominal ventrite of females (Fig. 87).

#### Males.

**Figures 74–82.**
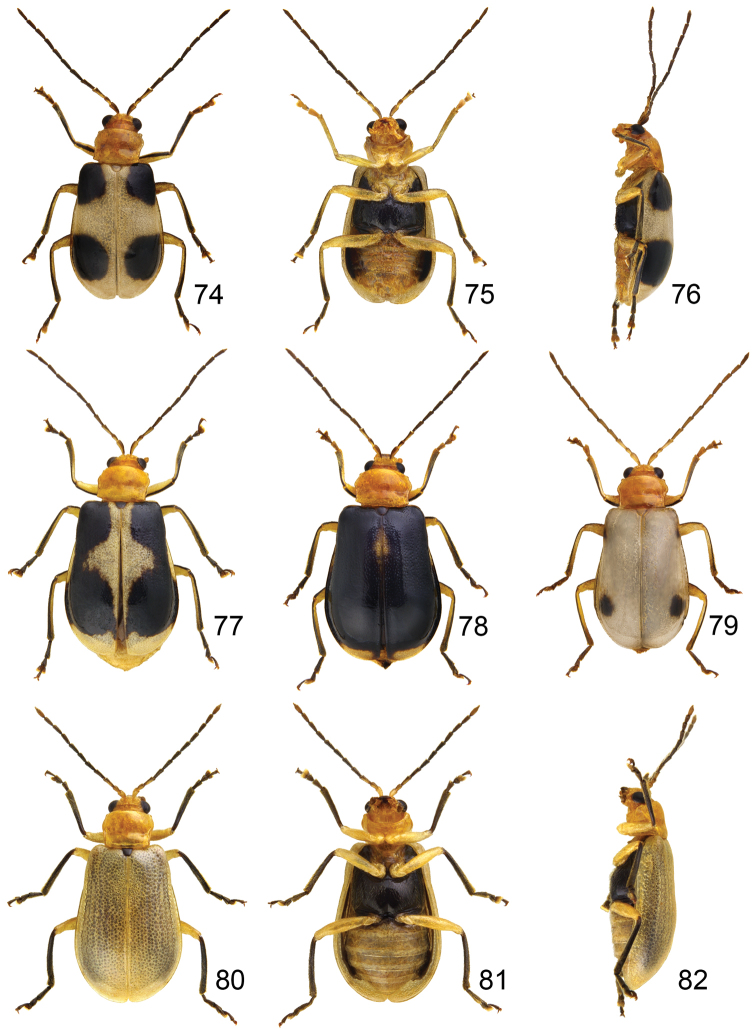
*Paridea* species. **74** *Paridea (Paridea) taiwana*, male, dorsal view **75** ditto, ventral view **76** ditto, lateral view **77** *Paridea (Paridea) taiwana*, black spots enlarged **78** *Paridea (Paridea) taiwana*, black elytra with yellow apices **79** *Paridea (Paridea) taiwana*, black spots reduced **80** *Paridea (Paridea) testacea*, female, dorsal view **81** ditto, ventral view **82** ditto, ventral view.

**Figures 83–89.**
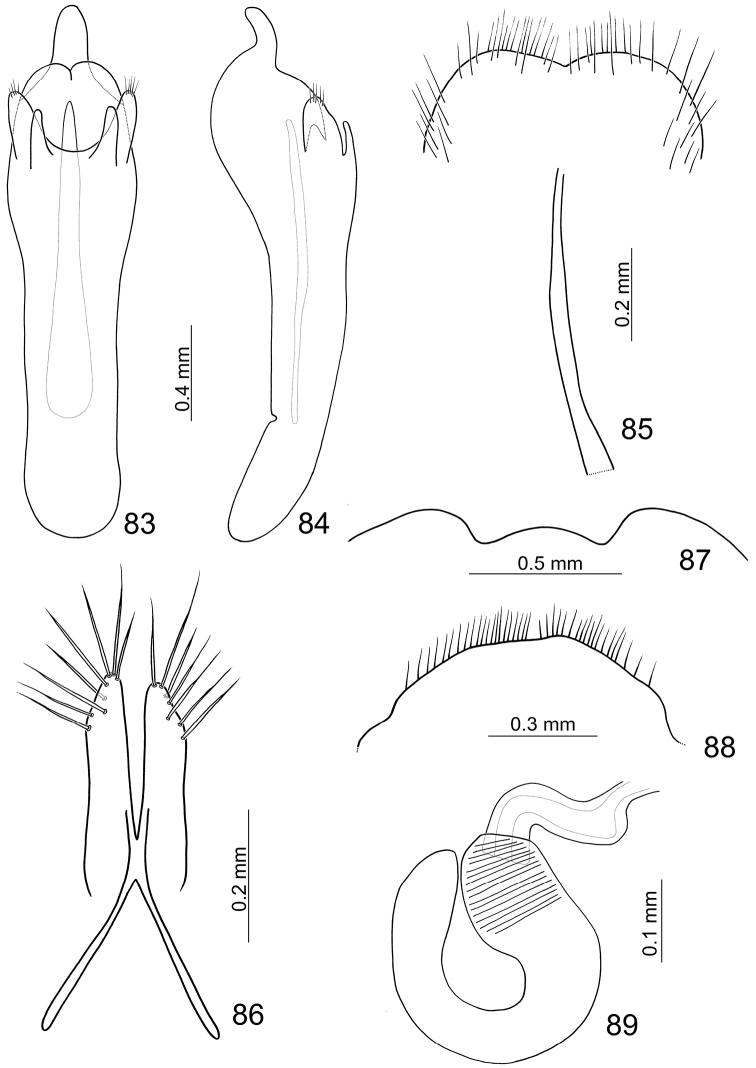
*Paridea (Paridea) taiwana*. **83** Penis, dorsal view **84** Penis, lateral view **85** Eighth abdominal sternite **86** Gonocoxae **87** Fifth abdominal ventrite **88** Eighth abdominal tergite **89** Spermatheca.

Length 5.1–5.9 mm, width 2.8–3.2 mm. General color (Figs 74–76) yellowish brown, two pairs of black spots on elytra, anterior one near humerus and other subapical, sizes of spots variable, sometimes spots enlarged and connected with each other (Fig. 77), even whole elytra black except apices (Fig. 78), sometimes black spots reduced (Fig. 79); outer margins of femora and tibiae black; metathoracic ventrites black. Eighth abdominal tergite (Fig. 88) weakly sclerotized, transverse and wide, apical margin slightly emarginate at middle, with dense long seta along apical margin. Penis (Figs 83–84) wide; apex tubular, curved from lateral view; strongly widened near apex from lateral view; external process small, smaller than medial process; medial process wide apex with several setae.

#### Females.

Length 4.8–7.1 mm, width 2.7–3.7 mm. Similar to male; apical margin of last abdominal ventrite emarginate at middle, slightly convex at emargination. Gonocoxae (Fig. 86) slender, apex of each gonocoxa with seven or eight setae from apical 1/7 to apex; connection of gonocoxae extremely slender, base slender. Sternite VIII (Fig. 89) weakly sclerotized; apex wide, apical margin concave at middle, surface with dense long setae along apical margin, spiculum short. Spermathecal receptaculum (Fig. 15) slightly swollen; pump extremely long, strongly curved; spermathecal duct short, stout, shallowly projecting into receptaculum.

#### Distribution.

Endemic to Taiwan. It is the most common and widespread species of the genus in Taiwan (Fig. 66).

#### Host plant.

Cucurbitaceae: *Thladiantha nudiflora* Hemsl. ex Forbes & Hemsl.

### Paridea (Paridea) testacea

Gressitt & Kimoto, 1963

http://species-id.net/wiki/Paridea_testacea

Paridea (Paridea) testacea Gressitt & Kimoto, 1963: 515; Kimoto 1969: 33; Kimoto 1989a: 251; Kimoto 1991: 11.
Paraulaca flavipennis Chûjô, 1935: 165 (nec Laboissière 1930: 334); Chûjô 1938: 138; Kimoto 1966: 29 (as synonym of *Paridea testacea*). synonym confirmed
Paraulaca (Paraulaca) flavipennis : Chûjô 1962: 193 (redescription); Chûjô 1965: 97.
Paridea (Paridea) formosana Yang, 1991: 272. (replacement name for *Paraulaca flavipennis*)

#### Type locality.

China, Fujian, Shaowu, Tachulan.

#### Type material examined.

*Paridea (Paridea) testacea*: Holotype female (BPBM), pinned, labeled: “Fukien, S. China [p] / Shawu, TaChu[p]Lan [h] / III-29-42 [h] T. C. Maa [p, w] // HOLOTYPE [p] ♂ [h] / *Paridea* [h] / (*Paridea*) [h] / *testacea* [h] / Gressitt & Kimoto [p, r] // *Paridea (P.)* [h] / *testacea* [h] / sp. 3 [h] / holo [h] / Det. S. Kimoto [p, w]”. Gressitt and Kimoto (1963) indicated that the holotype is a male. Actually it is a female. Paratypes: 1♀: “Fukien, S. China [p] / Shaowu, Tachulan [p] / IV.11.[h]194[p]3[h] T. Maa [p, w] // T. C. Maa, collec- [p] / tor. L. Gressitt [p] / collection [p, w] // allo [h] // *Paridea* [h] / *testacea* [h] / ♀ G & K [h] / Gressitt & Kimoto det. 196[p]1[h, w] // ALLOTYPE [p] / *Paridea* [h] / *testacea* [h] / S. Kimoto [h] / J. L. Gressit [p, r]; 1♀ (BPBM): “Fukien S. China [p] / ShaoWu TaChuFung [h] / IV-24-[h],19[p]42[h] T.C.Maa [p, w] // PARATYPE [p] / *Paridea* [h] / *testacea* [h] / Gressitt & Kimoto [p, y] // *Paridea* [h] / *testacea* [h] / G & K [h] / Gressitt & Kimoto det. 1961 [p, w]”; 1♀ (BPBM): “Fukien. S. China [p] / Shaowu, Tachulan [p] V.31[h].194[p]2[h] T. Maa [p, w] // T.C.Maa, collec- [p] tor. L.Gressitt [p] / collection [p] // *Paridea* [h] / *testacea* [h] / G & K [h] / Gressitt & Kimoto det. 1961 [p, w]”; 1♀ (CAS): “FUKIEN, S. China [p] / Shaowu: Tachulan [p] / 1000 m. T. Maa [p, w] // Apr. 8, 1943 [h, w] // NO 6 [p, w] // PARATYPE [p] / *Paridea* [h] / *testacea* [h] / Gressitt & Kimoto [p, y] // *Paridea* [h] / *testacea* [h] / G & K [h] / Gressitt & Kimoto det. 1961 [p, w]”; 1♀ (CAS): “FUKIEN, S. China [p] / Shaowu: Tachulan [p] / 1000 m. T. Maa [p, w] // Apr. 17, 1943 [h, w] // PARATYPE [p] / *Paridea* [h] / *testacea* [h] / Gressitt & Kimoto [p, y] // *Paridea* [h] / *testacea* [h] / G & K [h] / Gressitt & Kimoto det. 1961 [p, w]”; 1♀ (BPBM): “FUKIEN, S. China [p] / Shaowu: Tachulan [p] / 1000 m. T. Maa [p, w] // APR. 27, 1943 [h, w] // *Paridea* [h] / *testacea* [h] / G & K [h] / Gressitt & Kimoto det. 196 [p] 1 [h, w] // PARATYPE [p] / *Paridea* [h] / *testacea* [h] / Gressitt & Kimoto [p, y]”; 1♀ (CAS): “FUKIEN, S. China [p] / Shaowu: Tachulan [p] / 1000 m. T. Maa [p, w] // V-3-1943 [h, w] // PARATYPE [p] / *Paridea* [h] / *testacea* [h] / Gressitt & Kimoto [p, y]”; 1♀ (CAS): “Fukien, S. China [p] / Shaowu, TaChuFung May. 6–10. 1943 [p] T. C. Maa [p, w] // PARATYPE [p] / *Paridea* [h] / *testacea* [h] / Gressitt & Kimoto [p, y] // *Paridea* [h] / *testacea* [h] / G & K [h] / Gressitt & Kimoto det. 1961 [p, w]”, although the locality of this type is different locality from that of holotype, it is still regarded as paratypes since collecting date was included in the original description and bearing the same type label; 1♀ (BPBM), same data as preceding; 1♀ (BPBM): “Fukien, S. China [p] / Shaowu, TaChuFung [p] / V [h] / 26-29[h]-43. T. C. Maa [p, w] // PARATYPE [p] / *Paridea* [h] / *testacea* [h] / Gressitt & Kimoto [p, y]”; 1♀ [BPBM]: “FUKIEN, S. China [p] / Shaowu: TaChuLan [p] / K.S. Lin [h] T. C. Maa [p, w] // PARATYPE [p] / *Paridea* [h] / *testacea* [h] / Gressitt & Kimoto [p, y] // *Paridea* [h] / *testacea* [h] / G & K [h] / Gressitt & Kimoto det. 1961 [p, w]”; 1♀ (BPBM): “Fukien. S. China [p] / Shaowu, Tachulan [p] / VI.2[h].194[p]3[h] T. Maa [p, w] // T.C. Maa, collec- [p] / tor. L. Gressitt [p] / collection [p, w] // PARATYPE [p] / *Paridea* [h] / *testacea* [h] / Gressitt & Kimoto [p, y] // *Paridea* [h] / *testacea* [h] / G & K [h] / J.L.Gressitt det. [p, w]”.

*Paraulaca flavipennis*: Lectotype male (TARI), pinned, here designated to fix the concept of *Paraulaca flavipennis* Chûjô and to ensure the universal and consistent interpretation of the same, labeled: “Formosa [p] / Arisan [= Alishan, in Chiayi], 1918. [p] / X 2-23. [p] / J. Sonan. [p, w] // COTYPE [p, circle label with yellow letters] // *Paraulaca* [h] / *flavipennis* [h] / Chûjô [h] / DET. M. CHUJO [p, g] // 2177 [p, w] // **Lectotypus** [p] / *Paraulaca flavipennis* ♂ [p] / Chûjô, 1935 [p] / des. C.-F. Lee, 2014 [p, r]”. Paralectotypes: 1♀, same as lectotype but with “2178”; 1♂ (TARI): “Arisan [=Alishan, in Chiayi] [h] / 1912.X.10 [h] / Col. I. Nitobe [p, w] // COTYPE [p, circle label with yellow letters] // *Paraulaca* [h] / *flavipennis* [h] / Chûjô [h] / DET. M. CHUJO [p, g] // one kept [h] / (not paratype) [h, w]”; 1♂ (TARI): “Arisan [= Alishan, in Chiayi] [p] / FORMOSA [p] / 25.X.1933 [p] / Col. M. CHUJO [p] // COTYPE [p, circle label with yellow letters] // *Paraulaca* [h] / *flavipennis* [h] / Chûjô [h] / DET. M. CHUJO [p, g] // No. 1345 [p, w]”; 1♂, 1♀ [TARI]: “Formosa [p] / Karenko [= Hualien], -19. [p] / VII 20-VIII 4. [p] / T. Okuni, [p] // COTYPE [p, circle label with yellow letters] // *Paraulaca* [h] / *flavipennis* [h] / Chûjô [h] / DET. M. CHUJO [p, g] // 2180 and 2181 [p, w]”; 1♀ (TARI): “Formosa [p] / Musha [= Wushe, in Nantou]. 1919 [p] / V 18 – VI 15 [p] / T. Okuni, [p, w] // COTYPE [p, circle label with yellow letters] // *Paraulaca* [h] / *flavipennis* [h] / Chûjô [h] / DET. M. CHUJO [p, g] // 2179 [p, w]”; 1♂, 1♀ (TARI): “Formosa [p] / Shinchiku [= Hsinchu], -18. [p, w] / VII 1-30. [p] / J. Sonan, [p, w] // COTYPE [p, circle label with yellow letters] // *Paraulaca* [h] / *flavipennis* [h] / Chûjô [h] / DET. M. CHUJO [p, g)] // 2175 and 2176 [p, w]”; 1♂ (TARI): “Formosa [p] / Y. Miwa [p] // 西村[= Hsitsun, in Maoli] [h] / 24.7.1929 [h, w] [on the back] // COTYPE [p, circle label with yellow letters] // *Paraulaca* [h] / *flavipennis* [h] / Chûjô (h) / DET. M. CHUJO [p, g] // 757 [p, w]”; 1♀ (TARI): “Formosa [p] / Y. Miwa [p] // Hsuangyuang (in Taoyuan) [written in Japanese] [h] / 23.7.1929 [h, w] [on the back] // COTYPE [p, circle label with yellow letters] // *Paraulaca* [h] / *flavipennis* [h] / Chûjô [h] / DET. M. CHUJO [p, g] // 2173 [p, w]”; 1♂ (TARI): “Piyasan [written in Japanese] [in Taoyuan] [h] / VII. 1933 [h] / R. takahashi [written in Japanese] [h] // COTYPE [p, circle label with yellow letters] // *Paraulaca* [h] / *flavipennis* [h] / Chûjô [h] / DET. M. CHUJO [p, g] // 2172 [p, w]”; 1♀ (TARI): “Fukiko [= Fenchihu, in Chiayi] [p] / 29-IV-1931 [p] / Col. T. Shiraki [p, w] // COTYPE [p, circle label with yellow letters] // *Paraulaca* [h] / *flavipennis* [h] / Chûjô [h] / DET. M. CHUJO [p, g] // 2171 [p, w]”; 1♂ (TARI): “Jujiro [= Shihtzulu, in Chiayi] [p] / 26-IV-1931 [p] / Col. T. Shiraki [p, w] // COTYPE [p, circle label with yellow letters] // *Paraulaca* [h] / *flavipennis* [h] / Chûjô [h] / DET. M. CHUJO [p, g] // 2170 [p, w]”; 1♂, 1♀ (SDEI): “Taihorin [= Talin, in Chiayi] [p] / Formosa [p] / H. Sauter, 1911 [p, w] // 7. VII [p, w] // Syntypus [p, r] // *Paraulaca* [h] / *flavipennis* [h] / Chûjô [h] / DET. M. CHUJO [p, g] // DEI Müncheberg [p] / Col – 04213 and 04214 [p, g]”. Each paralectotype has a type label: “**Paralectotypus** [p] / *Paraulaca flavipennis* ♂ [or ♀] [p] / Chûjô, 1935 [p] / des. C.-F. Lee, 2014 [p, pink label]”.

#### Specimens examined

**(n = 391).** **CHINA:** **Fujian:** 1♂, Shaowu, Tachulan, 5.IV.1942, leg. T. C. Maa (BPBM); **TAIWAN: Hsinchu:** 1♀, Chienshih, 26.IX.2009, leg. H.-J. Chen (TARI); 1♀, Kuanwu, 30.IV.2010, leg. C.-F. Lee (TARI); 7♂♂, 1♀, same locality, 4.III.2010, leg, L.-H. Sun (TARI); 1♂, 1♀, Litungshan, 15.III.2009, leg. S.-F. Yu (TARI); 1♀, same locality, 6.VI.2010, leg. Y.-L. Lin (TARI); 1♂, same locality, 10.VII.2010, leg. Y.-L. Lin (TARI); 1♀, Lupi, 26.VII.2008, leg. M.-H. Tsou (TARI); 11♀♀, Mamei, 4.V.2008, leg. S.-F. Yu (TARI); 1♂, Peitelaman, 26.VI.2008, leg. S.-F. Yu (TARI); 1♂, Tahunshan, 8.IX.2009, leg. S.-F. Yu (TARI); 1♂, Wuchihshan, 27.III.2008, leg. H. Lee (TARI); 1♂, Wufeng, 17.III.2009, leg. S.-F. Yu (TARI); **Ilan:** 1♀, Taipingshan, 26–28.VII.1983, leg. L. Y. Chou (TARI); **Kaoshiung:** 2♂♂, Erhchituan, 21.VI.2010, leg. U. Ong (TARI); 3♀♀, same locality, 8.III.2013, leg. B.-X. Guo (TARI); **Miaoli:** 1♂, Kuantaoshan, 3.XI.2009, leg. S.-F. Yu (TARI); 1♂, same locality, 5.II.2012, leg. M.-H. Tsou (TARI); 1♂, Sanyi, 6.VII.2013, leg. Y.-T. Chung (TARI); **Nantou:** 1♂, Chingching, 5.III.2007, leg. H.-J. Chen (TARI); 2♂♂, 1♀, same locality, 27.VII.2013, leg. W.-C. Liao (TARI); 2♂♂, 3♀♀, Hoshe, 22.VII.1982, leg. L. Y. Chou & T. Lin (TARI); 1♂, 1♀, Huakang, 14.IX.2010, leg. C.-F. Lee (TARI); 1♀, Nanshanchi, 11.VII.2007, leg. M.-H. Tsou (TARI); 1♀, Meifeng, 20–22.VI.1979, leg. K. S. Lin & B. H. Chen (TARI); 1♂, same locality, 29.VIII-10.IX.1979 (TARI); 1♂, same locality, 8–14.XI.1979 (TARI); 2♂♂, same locality, 2–12.X.1979 (TARI); 1♀, same locality, 24.X.1979, leg. K. C. Chou (TARI); 2♂♂, same locality, 25.X-7.XI.1979 (TARI); 1♂, 4♀♀, same locality, 5–9.X.1980, leg. C. C. Chen & C. C. Chien (TARI); 1♀, same locality, 7–9.V.1981, leg. K. S. Lin & S. C. Lin (TARI); 1♂, same locality, 28–29.VIII.1981, leg. L. Y. Chou & S. C. Lin (TARI); 1♂, 3♀♀, same locality, 7.XI.1981, leg. S. C. Lin & W. S. Tang (TARI); 1♂, same locality, 15.VII.1982, leg. S. C. Lin & C. N. Lin (TARI); 12♂♂, 9♀♀, same locality, 31.VIII-2.IX.1982, leg. L. Y. Chou & K. C. Chou (TARI); 1♂, 1♀, same locality, 4–7.X.1982, leg. K. C. Chou (TARI); 1♀, Rueiyan River Major Wildlife Habitat, 8.VIII.2008, leg. H. Medel & M. V. L. Barclay (BMNH); 3♂♂, 1♀, Sungkang, 13–15.IX.1984, leg. K. S. Lin & S. C. Lin (TARI); 1♂, 4.IV.2010, leg. Y.-T. Wang (TARI); 1♂, 4♀♀, Tsuifeng, 1–3.VIII.1981, leg. T. Lin & W. S. Tang (TARI); 1♂, same locality, 27.VIII.1981, leg. L. Y. Chou & S. C. Lin (TARI); 1♂, same locality, 1–3.IX.1982, leg. L. Y. Chou & K. C. Chou (TARI); 1♂, 4♀♀, Tungpu, 20–22.VI.1980, leg. C. C. Chen (TARI); 2♂♂, 23♀♀, same locality, 28.IV-2.V.1981, leg. T. Lin & C. J. Lee (TARI); 17♂♂, 10♀♀, same locality, 5–8.X.1981, leg. T. Lin & W. S. Tang (TARI); 10♂♂, 9♀♀, same locality, 18–23.XI.1981, leg. T. Lin & W. S. Tang (TARI); 27♂♂, 24♀♀, same locality, 19–23.VII.1982, leg. L. Y. Chou & T. Lin (TARI); 7♂♂, 2♀♀, same locality, 22–25.XI.1982, leg. K. C. Chou & S. P. Huang (TARI); 6♂♂, 5♀♀, same locality, 20–24.VI.1983, leg. K. C. Chou & C. Y. Wong (TARI); 1♂, 13♀♀, same locality, 16–20.IV.1984, leg. K. C. Chou & C. H. Yang (TARI); 27♂♂, 35♀♀, same locality, 23–27.VII.1984, leg. K. C. Chou & C. H. Yang (TARI); 1♂, Wanfengtsun, 2.IV.2008, leg. W.-T. Liu (TARI); 1♂, same locality, 13.IV.2010, leg. W.-T. Liu (TARI); 1♂, Wushe, 23–28.VI.1981, leg. K. S. Lin & W. S. Tang (TARI); 3♂♂, 6♀♀, 30.VIII-2.IX.1982, leg. L. Y. Chou & K. C. Chou (TARI); 1♂, 1♀, same locality, 7–8.X.1982, leg. K. C. Chou (TARI); 1♂, 2♀♀, same locality, 19–22.IV.1983, leg. K. C. Chou & S. P. Huang (TARI); 2♀♀, same locality, 7.V.1984, leg. K. C. Chou & S. P. Huang (TARI); 1♂, same locality, 4.VIII.1984, leg. K. S. Lin (TARI); 5♂♂, same locality, 17.VIII.1984, leg. K. C. Chou (TARI); 1♂, same locality, 11–15.IX.1984, leg. K. S. Lin (TARI); 4♂♂, 1♀, same locality, 21–23.III.2009, leg. U. Ong (TARI); **Pingtung:** 1♀, Shouka, 22.III.2009, leg. M.-H. Tsou (TARI); 1♀, Tahanshan, 18.VII.2007, leg. C.-F. Lee (TARI); **Taichung:** 2♂♂, 3♀♀, Kukuan, 16.VII.2007, leg. M.-H. Tsou (TARI); 2♂♂, 1♀, Lishan, 16.VIII.1984, leg. K. S. Lin & S. C. Lin (TARI); 4♀♀, Wushihkeng, 19.III.2008, leg. C.-F. Lee (TARI); 1♂, Yuantsuishan, 16.VII.2010, leg. J.-C. Chen (TARI); **Taipei:** 2♀♀, Guanyinshan, 14.-21.IV.2002, leg. G. Fabián & Merkl O. (JBCB, HNHM); **Taitung:** 4♂♂, Motien, 5.X.2010, leg. C.-F. Lee (TARI); 1♂, 1♀, same locality, 23.V.2011, leg. C.-F. Lee (TARI); **Taoyuan:** 2♂♂, 3♀♀, Hsuanyuan, 16.III.2008, leg. M.-H. Tsou (TARI); 1♂, Sankuang, 17.X.2009, leg. Y.-L. Lin (TARI); 1♂, Tamanshan, 2.VIII.2008, leg. M.-H. Tsou (TARI); 1♀, Tungyangshan, 12.IV.2007, leg. H. Lee (TARI).

#### Diagnosis.

The species is similar to a few individuals of *Paridea (Paridea) taiwana* having reduced black spots on the elytra but differs by the yellow femora and black tibiae (black outer margins of femora and tibiae in *Paridea (Paridea) taiwana*).

#### Males.

**Figures 90–95.**
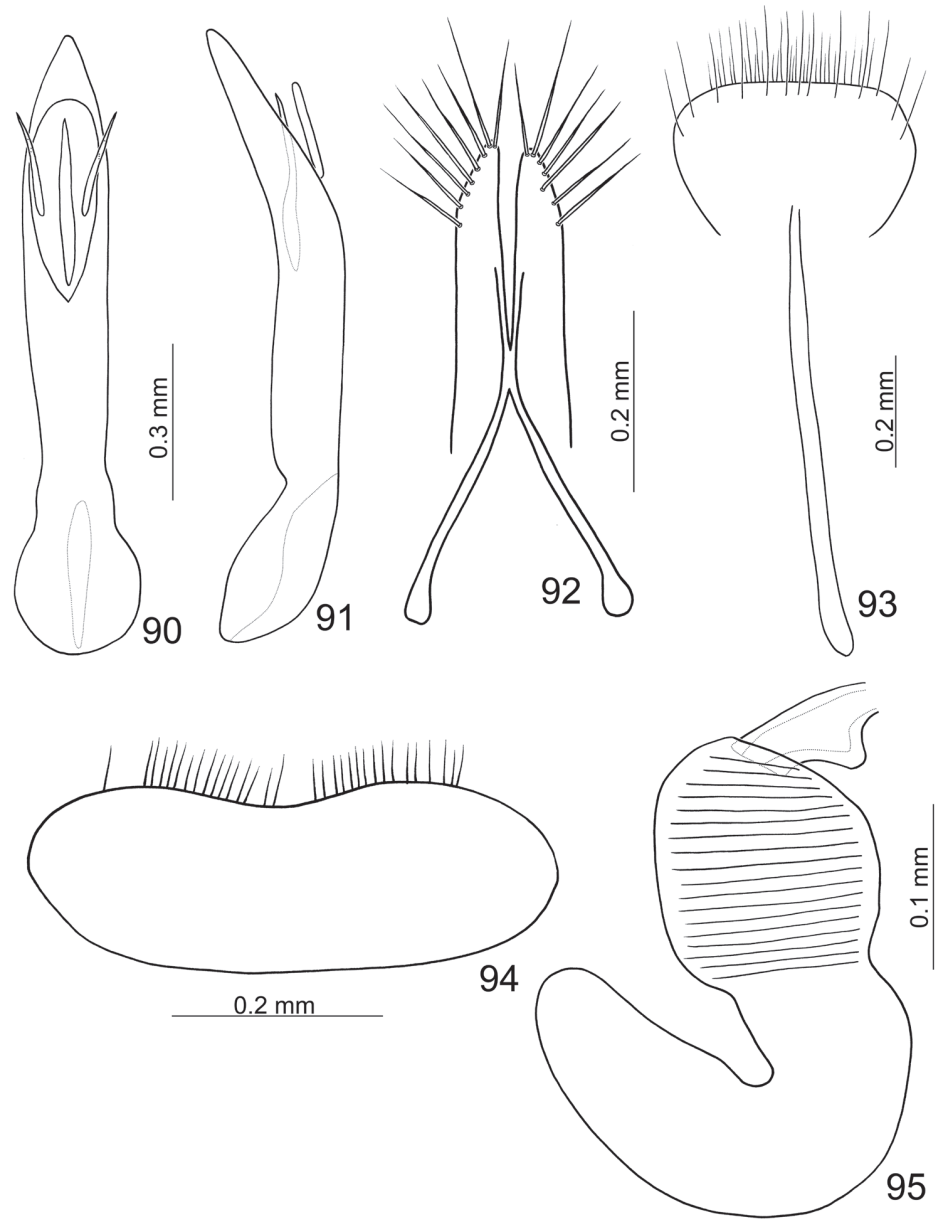
*Paridea (Paridea) testacea*. **90** Penis, dorsal view **91** Penis, lateral view **92** Gonocoxae **93** Eighth abdominal sternite **94** Eighth abdominal tergite **95** Spermatheca.

Length 4.4–4.6 mm, width 2.4–2.5 mm. General color (Figs 80–82) pale yellow; antenna brown; scutellum, tibia, and tarsi blackish brown; mesepimeron and metathoracic ventrites black. Eighth abdominal tergite (Fig. 94) weakly sclerotized, transverse and wide, apical margin slightly emarginate at middle, with dense long seta along apical margin. Penis (Figs 90–91) slender, apically pointed; slightly curved at middle in lateral view; with one pair of elongated sclerites projecting beyong opening; endophallic sclerites composed of one slender sclerite.

#### Females.

Length 4.6–5.3 mm, width 2.4–2.8 mm. Similar to male; apical margin of last abdominal ventrite smooth, not modified. Gonocoxae (Fig. 92) slender, apex of each gonocoxa with seven or eight setae from apical 1/7 to apex; connection of gonocoxae extremely slender, base slender. Sternite VIII (Fig. 93) weakly sclerotized; apex wide, apical margin truncate, surface with dense long setae along apical margin, spiculum long. Spermathecal receptaculum (Fig. 95) slightly swollen; pump long, strongly curved; spermathecal duct short, stout, shallowly projecting into receptaculum.

#### Host plants.

Cucurbitaceae: *Thladiantha nudiflora* Hemsl. ex Forbes & Hemsl.

#### Distribution.

China (Fujian), China. This species is common and widespread in Taiwan (Fig. 67).

#### Notes.

Taiwanese populations have a black scutellum which differs from the holotype *Paridea testacea* with a yellowish brown scutellum. Actually, most of types of *Paridea testacea* have the scutellum darkened. In addition, all of the studied types of *Paridea testacea* (including holotype and allotype) are females. One male was found from Maa’s collection at the BBPM. Examination of the male confirms that both are conspecific.

### Species excluded from Taiwan fauna

#### Paridea (Semacia) angulicollis

(Motschulsky, 1854)

http://species-id.net/wiki/Paridea_angulicollis

Rhaphidopalpa angulicollis Motschulsky, 1854: 50.
Aulacophora angulicollis : Baly 1874: 186.
Paraulaca (Aulacophora) angulicollis : Baly 1888: 168.
Paraulaca (Semacia) angulicollis : Ogloblin 1936: 168.
Semacia (Semacia) angulicollis : Chûjô and Kimoto 1961: 168.
Paridea (Paraulaca) angulicollis : Gressitt and Kimoto 1963: 508; Kimoto 1965b: 376.
Paridea (Paridea) angulicollis : Yang 1991: 269.
Semacia nipponensis Laboissière, 1930: 355; Chûjô and Kimoto 1961: 168 (as synonym of *Paridea angulicollis*).
Paridea (Semacia) nigrimarginata Yang, 1991: 279. syn. n.

##### Type locality.

China, Beijing.

##### Type material.

*Rhaphidopalpa angulicollis*: Unavailable for study. The type specimens are not present in the Zoological Museum of Moscow State University (Medvedev 2006, 2014, personal communication) or in the Zoological Institute in Saint Petersburg (Moseyko 2014, personal communication).

*Semacia nipponensis*: Lectotype male (MNHN), pinned, here designated to fix the concept of *Semacia nipponensis* and to ensure the universal and consistent interpretation of the same, labeled: “MUSEUM PARIS [p] / NIPPON MOYEN [p] / ENV. DE TOKIO [p] / ET ALPES DE NIKKO [p] / J. HARMAND 1901 [p, w] // *Semacia* [h] / *nipponensis* [h] / *m.* [h] / V. Laboissière – Dét. [p, w] // TYPE [red letters, p, w] ♂ [inserted between “Y” and “ P”, h] // SYNTYPE [p] / *Semacia* [p] / *nipponensis* Laboissière, 1930 [p, w] // SYNTYPE [p, r] // MNHN [p] / EC4060 [p, w] // **Lectotypus** [p] / *Senacua nipponensis* ♂ [p] / Laboissière, 1930 [p] / des. C.-F. Lee, 2014 [p, r]”. Paralectotypes: 1♂ (MNHN): “MUSEUM PARIS [p] / NIPPON MOYEN [p] / ENV. DE TOKIO [p] / ET ALPES DE NIKKO [p] / J. HARMAND 1901 [p, w] // SYNTYPE [p] / *Semacia* [p] / *nipponensis* Laboissière, 1930 [p, w] // SYNTYPE [p, r] // MNHN [p] / EC4061 [p, w]”; 1♀ (ZMUH): “Tokio [h, w] // TYPE [red letters, p, w] ♀ [inserted between “Y” and “P”, h] // *Semacia* [h] / *nipponensis* m [h] / V. Laboissière – Dét. [p, w]”. Each paralectotype has a type label: “**Paralectotypus** [p] / *Semacia nipponensis* ♂ [or ♀] [p] / Laboissière, 1930 [p] / des. C.-F. Lee, 2014 [p, pink label]”

*Paridea (Semacia) nigrimarginata*: Holotype ♂ (IZAS), labeled: “Mt. Takao [p] / June 11 32 [blue letters, p, w] // HOLOTYPE [p, r] // *Paridea (S.)* [h] / *nigrimarginata* [h] / sp n [h] / 鑑定者 [p]: 楊.[h, w] [= det. Yang]”.

##### Additional material examined

**(n = 14).** **CHINA.** **Jiangxi:** 1♂ (BPBM); **Hubei** (= Hupeh): 1♂, Lichuan, Lianghoken, 7.IX.1948, leg. Gressitt & Djou (BPBM); **Sichuan:** 2♂♂, Bayueshan, 21.IV.2013, leg. J. Y. Qiu & H. Xu (TARI); **JAPAN. Honshu:** 1♀ (KMNH), Aomori Pref., Towada, 20.VII.1980, leg. S. Kawauchi; 1♀ (KMNH), Kanagawa Pref., Tanzawa, Ooyma, 23.V.1966, leg. Y. Kusui; 1♂ (CAS), Tokyo, 27.IV.1930, leg. L. Gressitt; 1♀ (CAS), Tokyo Pref., Mt. Takao, 4.V.1930, leg. L. Gressitt; 1♀ (CAS), same locality, 14.VI.1959, leg. H. Toshimii; 1♀ (KMNH), Kyoto Pref., Minoo – Takayama, 8.V.1956, leg. K. Morimoto; **Kyushu:** 3♂♂ (KMNH), Fukuoka Pref., Mt. Sefuri, 10.VI.1956, leg. H. Kamiya; 1♂ (KMNH), Fukuoka Pref., Kurokidaira, 23.V.1979.

##### Diagnosis.

See diagnosis of *Paridea (Semacia) kaoi* sp. n.

##### Males.

**Figures 96–101.**
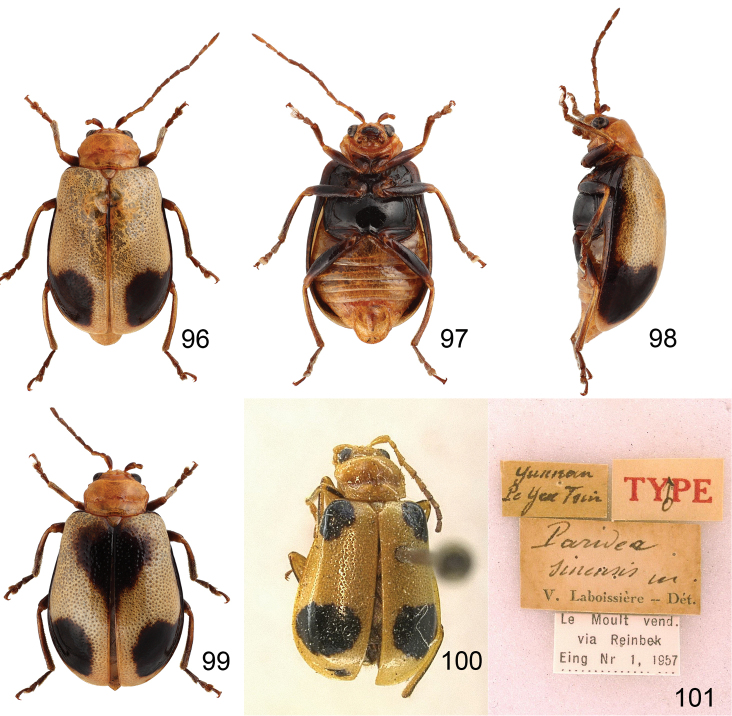
*Paridea* species. **96** *Paridea (Semacia) angulicollis*, male, dorsal view **97** ditto, ventral view **98** ditto, lateral view **99** *Paridea (Semacia) angulicollis*, female, dorsal view **100** *Paridea (Paridea) sinensis*, lectotype, dorsal view **101** *Paridea (Paridea) sinensis*, lectotype, labels.

Length 5.1–5.5 mm, width 3.1–3.3 mm. Head and prothorax yellowish brown (Figs 96–98), labrum black, antenna brown; scutellum pale yellow; elytra pale yellow, with deep excavation behind scutellum at suture; with one extremely slender black stripe along suture behind excavation, sometimes reduced; with one pair of large black spots subapically, lateral margin and epipleuron black, extending posterior and connected with subapical black spots; meso- and metathoracic ventrites black; legs dark brown, apex of femur and base of tibia paler; abdomen yellow. Eighth abdominal tergite (Fig. 107) strongly sclerotized, transverse and slender, with one pair of extremely slender and curved processes. Pygidium slightly projecting beyong elytral apices. Penis slightly asymmetric, slightly narrowed at apical 1/6; apex narrow, tubular, and small; straight from lateral view; endophallic sclerites with one pointed sclerite, one elongate sclerite, and a cluster of large setae.

##### Females.

Lenth 5.1–5.6 mm, width 3.0–3.4 mm. Similar to male (Fig. 99), elytra without excavation but black spot instead. Apical margin of last abdominal ventrite (Fig. 106) with one pair of small rounded processes at middle, slightly emarginate outside processes. Pygidium slightly projecting beyong elytral apices. Gonocoxae (Fig. 104) slender, apex of each gonocoxa with eight setae from apical 1/7 to apex; connection of gonocoxae extremely slender, base widened. Sternite VIII (Fig. 105) weakly sclerotized; apex narrow, apical margin emarginate at middle, surface with dense long setae along apical margin, spiculum short. Spermathecal receptaculum (Fig. 108) slightly swollen; pump much longer than receptaculum, strongly curved; spermathecal duct short, stout, shallowly projecting into receptaculum.,

**Figures 102–108.**
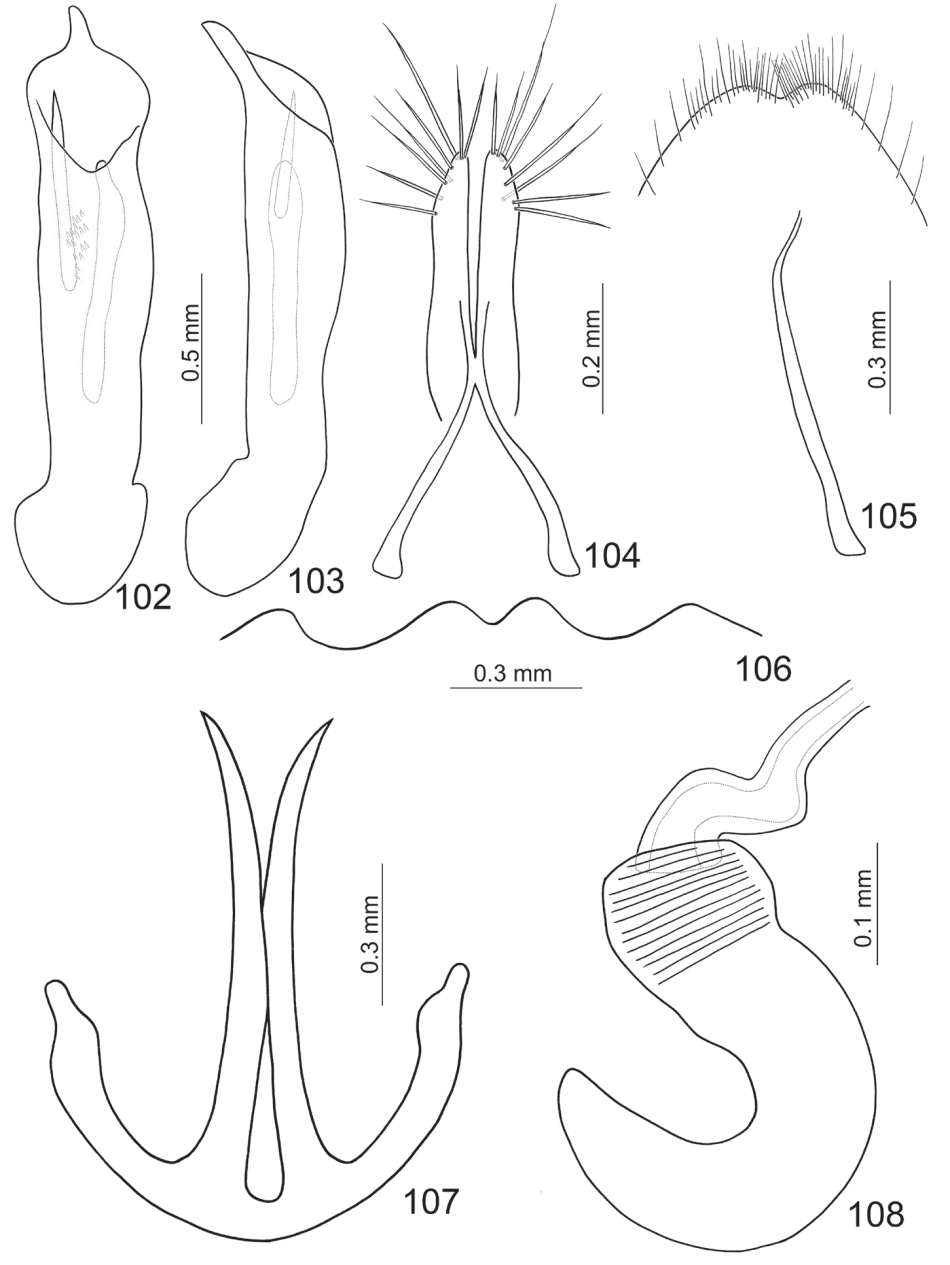
*Paridea (Semacia) angulicollis*. **102** Penis, dorsal view **103** Penis, lateral view **104** Gonocoxae **105** Eighth abdominal sternite **106** Fifth abdominal ventrite **107** Eighth abdominal tergite **108** Spermatheca.

##### Distrbution.

Japan, China (Jiangxi, Hubei, Sichuan). Yang (1991) indicated that this species was also found in Zhejiang, Fujian, Hainan, and Guanxi provinces of China. These records are dubious since no voucher specimens were examined and Japanese populations were misidentified as *Paridea (Semacia) nigrimarginata* Yang, 1991 (see below).

##### Host plants.

Cucurbitaceae: *Gynostemma pentaphyllum* (Thunb.) Makino, *Trichosanthes cucumeroides* (Ser.) Maxim. ex Fr. & Sav. (Chûjô and Kimoto 1961).

##### Notes.

The position of the type locality of *Paridea (Semacia) nigrimarginata* was doubtful since information on the label is insufficient. Only “Mt. Takao” appears on the label and Beenen (2010) supposed that it was located in Taiwan since “Takao” is the Japanese name for Kaoshiung City, but Kaoshiung city it is not a mountain. Mt. Takao probably refers to a locality in Japan since a famous mountain (Mt. Takao-Yama, Takao-machi, Hachioji-shi, Tokyo Prof., Japan) exists there with similar names. Moreover, subsequent material (see specimens examined) came from this locality with additional information indicating the Japanese Mt. Takao.

#### Paridea (Paridea) sinensis

Laboissière, 1930

http://species-id.net/wiki/Paridea_sinensis

Paridea sinensis Laboissière, 1930: 342.
Paridea (Paridea) sinensis : Gressitt and Kimoto 1963: 514.

##### Type locality.

China, Yunnan, PeYenTsin.

##### Type material.

Lectotype male (Fig. 100) (ZMUH), pinned, here designated to fix the concept of *Paridea sinensis* and to ensure the universal and consistent interpretation of the same, labeled (Fig. 101): “Yunnan [h] / Pe Yen Tsin [h, w] // TYPE [red letters, p] ♂ [p] // *Paridea* [h] / *sinensis* [h] / *m* [h] / V. Laboissière – Dét. [p, w] // Le Moult Vend. [p] / via Reinbek [p] / Eing Nr. 1, 1957 [p, w] // **Lectotypus** [p] / *Paridea sinensis* ♂ [p] / Laboissière, 1930 [p] / des. C.-F. Lee, 2014 [p, r]”. Paralectotypes: 1♀ (ZMUH): “Yunnan [h] / Pe Yen Tsin [h, w] // Le Moult Vend. [p] / via Reinbek [p] / Eing Nr. 1, 1957 [p, w]”; 1♀ (ZMUH): “**PE YEN TSIN** [p] / **YUNNAN** [p] / Coll. de Touzalin [p, w] // Le Moult Vend. [p] / via Reinbek [p] / Eing Nr. 1, 1957 [p, w]”; 1♀ (ISNB): “**PE YEN TSIN** [p] / **YUNNAN** [p] / Coll. de Touzalin [p, w], glued on larger card, labeled: “*Coll. R. I. Sc. N. B.* (p) / Chine [p, y] // *Paridea* [h] / *sinensis* [h] / m [h[ / V. Laboissière – Dét. [p] / 1930 [vertical, h, w] // Para- [p] / type [p, o]”; 1♀ (MNHN): “MUSEUM PARIS [p] / YUNNAN [p] / S.-O 24 ◦ N [p] / PE. YEN. TSIN [p] / (MINES DE SEL) [p] / (PÈRE SIMÉON TEN) [p] / P. CUERRY 1924 [p, w] // NOVEMBRE [p, w] // SYNTYPE [p] / *Paridea (Paridea)* [p] / *sinensis* Laboissière, 1930 [p, w] // SYNTYPE [p, r] / MNHN [p] / EC4062 [p, w]”; 1♀ (MNHN): “MUSEUM PARIS [p] / KOUY-TCHÉOU [p] / RÉG. DE PIN-FA [p] / PÈRE CAVALERIE 1908 [p, w] // SYNTYPE [p] / *Paridea (Paridea)* [p] / *sinensis* Laboissière, 1930 [p, w] // SYNTYPE [p, r] / MNHN [p] / EC4063 [p, w]”; 1♀ (MNHN): “MUSEUM PARIS [p] / SE-TCHOUEN [p] / ENV DE TA-TSIEN-LOU [p] / MO-SY-MIEN [p] / Père AUBERT 1902 [p, w] // *Paridea* [h] / *sinensis* [h] / m [h] / V. Laboissière – Dét. [p, w] // COTYPE [red letters, p, w] // SYNTYPE [p] / *Paridea (Paridea)* [p] / *sinensis* Laboissière, 1930 [p, w] // SYNTYPE [p, r] / MNHN [p] / EC4064 [p, w]”; 2♀♀ (MNHN): “MUSEUM PARIS [p] / SE-TCHOUEN [p] / ENV DE TA-TSIEN-LOU [p] / MO-SY-MIEN [p] / Père AUBERT 1902 [p, w] // SYNTYPE [p] / *Paridea (Paridea)* [p] / *sinensis* Laboissière, 1930 [p, w] // SYNTYPE [p, r] / MNHN [p] / EC4065 or 4066 [p, w]”. Each paralectotype has a type label: “**Paralectotypus** [p] / *Paridea sinensis* ♂ [or ♀] [p] / Laboissière, 1930 [p] / des. C.-F. Lee, 2014 [p, pink label]”

##### Additional material examined

**(n = 2).** **CHINA:** **Sichuan:** 1♂ (TARI), Luding, Moxi, 20.VI.1983, leg. Y.-Q. Chen; 1♀ (TARI), same but with “leg. S.-Y. Wang.

##### Diagnosis.

See diagnosis of *Paridea (Paridea) taiwana*.

##### Males.

**Figures 109–114.**
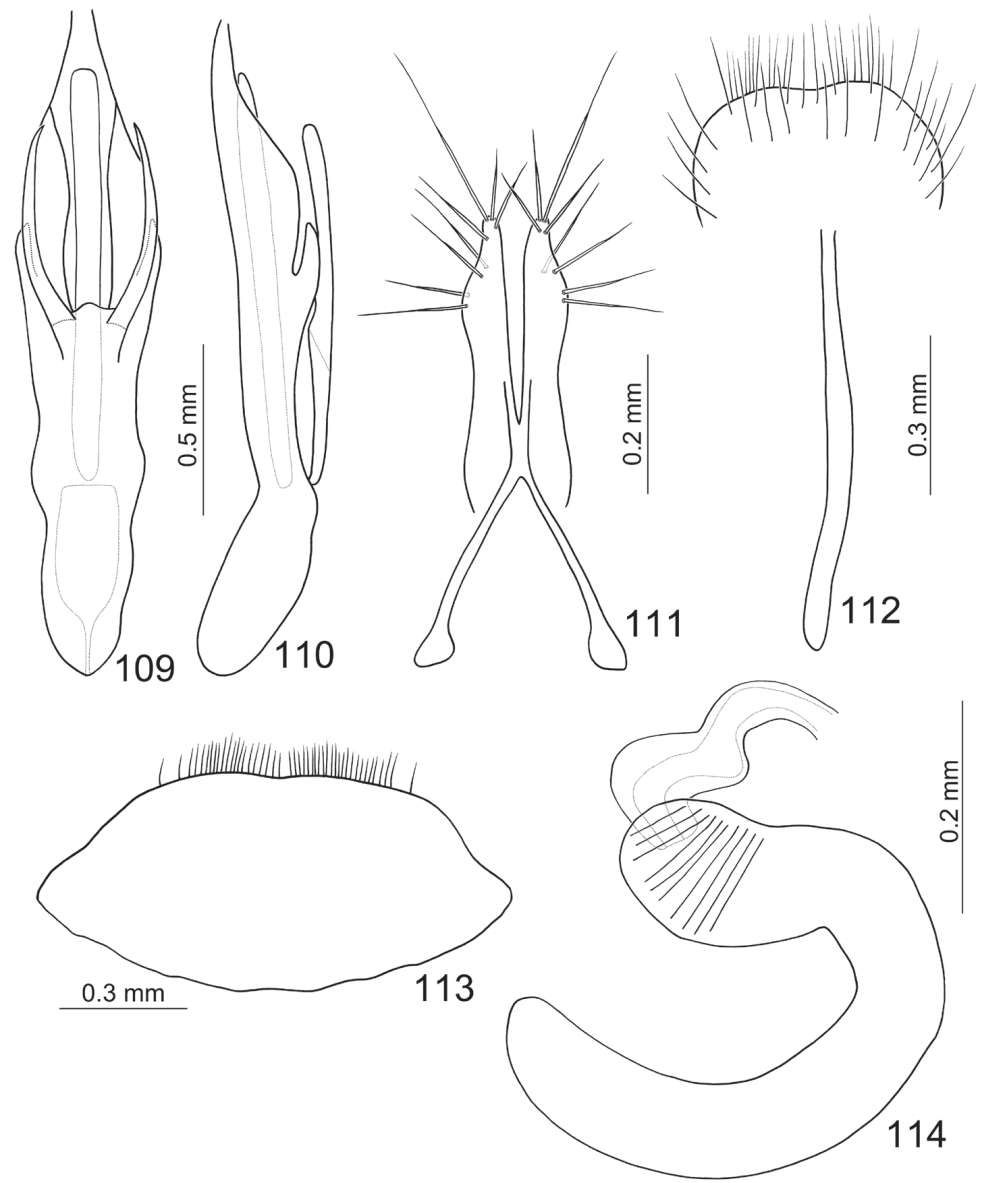
*Paridea (Paridea) sinensis*. **109** Penis, dorsal view **110** Penis, lateral view **111** Gonocoxae **112** Eighth abdominal sternite **113** Eighth abdominal tergite **114** Spermatheca.

Length 6.5 mm, width 3.6 mm. General color (Fig. 100) yellowish brown, elytra with two pairs of black spots: one at humerus smaller, other subapical and larger; outer margins of femora and tibiae black; metathoracic ventrites black. Eighth abdominal tergite (Fig. 113) weakly sclerotized, transverse and wide, apical margin slightly emarginate at middle, with dense long seta along apical margin. Penis (Fig. 109–110) slender; external process extending anterior, much longer than medial process; medial process small; straight in lateral view.

##### Females.

Length 6.8 mm, width 3.8 mm. Similar to male; apical margin of last abdominal ventrite smooth, not modified. Gonocoxae (Fig. 111) slender, apex of each gonocoxa with eight setae from apical 1/5 to apex; connection of gonocoxae extremely slender, base widened. Sternite VIII (Fig. 112) weakly sclerotized; apex narrow, apical margin slightly concave at middle, surface with longer setae near apical margin and shorter setae on apical margin, spiculum long. Spermathecal receptaculum (Fig. 114) slightly swollen; pump extremely long, strongly curved; spermathecal duct short, stout, shallowly projecting into receptaculum.

##### Distribution.

China (Jiangxi, Hubei, Fujiang, Sichuan, Yunnan, Guizhou). The distribution of *Paridea (Paridea) sinensis* should be reevaluated since specimens collected from Fujian identified by Gressitt & Kimoto are *Paridea (Paridea) fujiana* Yang, 1991.

## Supplementary Material

XML Treatment for Paridea (Semacia) houjayi

XML Treatment for Paridea (Semacia) kaoi

XML Treatment for Paridea (Semacia) sexmaculata

XML Treatment for Paridea (Paridea) costata

XML Treatment for Paridea (Paridea) cyanipennis

XML Treatment for Paridea (Paridea) sauteri

XML Treatment for Paridea (Paridea) taiwana

XML Treatment for Paridea (Paridea) testacea

XML Treatment for Paridea (Semacia) angulicollis

XML Treatment for Paridea (Paridea) sinensis
